# Supplementary material for: Extracting relevant predictive variables for COVID-19 severity prognosis: An exhaustive comparison of feature selection techniques
Source: PLoS One. 2023 Apr 13;18(4):e0284150. doi: 10.1371/journal.pone.0284150 (PMC10101453; doi:10.1371/journal.pone.0284150)
Supplement: S1 Appendix — Report—(S.A) Data: List of features, (S.B) Data: Cohort characteristics, (S.C) Methods: Hyperparameters, (S.D) Results: Stability, (S.E) Results: Computation times. (PDF) [file pone.0284150.s001.pdf]

On-line Supplementary Materials

S.A Data: List of features

Table S1. Numbered list of features in our dataset.

Num	Feature	Values or units	Type
0	Sex	Male, Female	Binary
1	Age	*[years]*	Continuous
2	Height	*[m]*	Continuous
3	Weight	*[kg]*	Continuous
4	Body mass index (BMI)	$[kg/m^2]$	Continuous
5	Obesity	No, Yes	Binary
6	Alcohol use	No = 0, Ex = 1, Yes = 2	Ordinal
7	Tobacco use	No = 0, Ex = 1, Yes = 2	Ordinal
8	Lives at nursing home	No, Yes	Binary
9	Comorbidity: Hypertension	No, Yes	Binary
10	Comorbidity: Diabetes mellitus	No, Yes	Binary
11	Comorbidity: Dyslipidemia	No, Yes	Binary
12	Comorbidity: Cardiovascular	No, Yes	Binary
13	Comorbidity: Chronic heart failure	No, Yes	Binary
14	Comorbidity: Cerebrovascular	No, Yes	Binary
15	Comorbidity: Broncho, Asthma	No, Yes	Binary
16	Comorbidity: Broncho, ILD	No, Yes	Binary
17	Comorbidity: Broncho, COPD	No, Yes	Binary
18	Comorbidity: Broncho, Others	No, Yes	Binary
19	Comorbidity: Renal	No, Yes	Binary
20	Comorbidity: Hepatic	No, Yes	Binary
21	Comorbidity: Ischemia	No, Yes	Binary
22	Comorbidity: Peptic ulcer	No, Yes	Binary
23	Comorbidity: Thyroid	No, Yes	Binary
24	Comorbidity: Autoimmune	No, Yes	Binary
25	Comorbidity: Transplants	No, Yes	Binary
26	Comorbidity: Cancer-neoplasia	No, Yes	Binary
27	Comorbidity: Immune-HIV	No, Yes	Binary
28	Comorbidity: Overall	No, Yes	Binary
29	Comorbidity: Charlson index	—	Integer
30	Pneumonia: CURB-65 score	—	Integer
31	Pneumonia: PSI score	—	Integer
32	Sepsis: qSOFA score	—	Integer
33	Symptoms: Cough	No, Yes	Binary
34	Symptoms: Expectoration	No, Yes	Binary
35	Symptoms: Dyspnea	No, Yes	Binary
36	Symptoms: Myalgia	No, Yes	Binary
37	Symptoms: Confusion	No, Yes	Binary
38	Symptoms: Thorax pain	No, Yes	Binary
39	Symptoms: Anosmia	No, Yes	Binary

Num	Feature	Values or units	Type
40	Symptoms: Fever	No = 0, Febricula = 1, Fever = 2	Ordinal
41	Symptoms: Digestive-gastro	No, Yes	Binary
42	Symptoms: Overall	No, Yes	Binary
43	Symptoms: Days	—	Integer
44	Emergency treatm: ACEI-AIIRB	No, Yes	Binary
45	Emergency treatm: Statin	No, Yes	Binary
46	Emergency treatm: Anticoagulant	No, Yes	Binary
47	Emergency treatm: Antiplatelet	No, Yes	Binary
48	Emergency treatm: Corticosteroids, Inhaled	No, Yes	Binary
49	Emergency treatm: Corticosteroids, Oral	No, Yes	Binary
50	Emergency treatm: Corticosteroid, Missing	No, Yes	Binary
51	Admission status: Body temperature	*[°C]*	Continuous
52	Admission status: Systolic blood pressure	*[mmHg]*	Continuous
53	Admission status: Diastolic blood pressure	*[mmHg]*	Continuous
54	Admission status: Respiratory rate	[min^{-1}]	Continuous
55	Admission status: Heart rate	[min^{-1}]	Continuous
56	Admission status: SpO_2	*[%]*	Continuous
57	Admission status: FiO_2	*[fraction]*	Continuous
58	Admission status: SpO_2/FiO_2	*[ratio]*	Continuous
59	Admission status: SpO_2/RespRate	[%/min^{-1}]	Continuous
60	Pulmonary status: Crackles	No, Yes	Binary
61	Pulmonary status: Infiltr. X-Ray, Unilobar	No, Yes	Binary
62	Pulmonary status: Infiltr. X-Ray, Multilobar unilateral	No, Yes	Binary
63	Pulmonary status: Infiltr. X-Ray, Bilateral	No, Yes	Binary
64	Pulmonary status: Infiltr. X-Ray, Missing	No, Yes	Binary
65	Pulmonary status: Infiltr. type, Alveolar	No, Yes	Binary
66	Pulmonary status: Infiltr. type, Consolidation	No, Yes	Binary
67	Pulmonary status: Infiltr. type, Interstitial	No, Yes	Binary
68	Pulmonary status: Infiltr. type, Missing	No, Yes	Binary
69	Pulmonary status: Infiltration, Num. lobes	—	Integer
70	Pulmonary status: Pleural effusion	No, Yes	Binary
71	Blood test: Glucose	*[mg/dL]*	Continuous
72	Blood test: Urea [$\log_{10}$]	*[mg/dL]*	Continuous
73	Blood test: Creatinine [$\log_{10}$]	*[mg/dL]*	Continuous
74	Blood test: Blood urea nitrogen (BUN) [$\log_{10}$]	*[mg/dL]*	Continuous
75	Blood test: Sodium	*[mEq/L]*	Continuous
76	Blood test: Gamma-glutamyl transferase (GGT) [$\log_{10}$]	*[U/L]*	Continuous
77	Blood test: Lactate dehydrogenase (LDH) [$\log_{10}$]	*[U/L]*	Continuous
78	Blood test: C-Reactive protein (CRP) [$\log_{10}$]	*[mg/L]*	Continuous
79	Blood test: Procalcitonin (PCT) [$\log_{10}$]	*[µg/L]*	Continuous
80	Blood test: Hematocrit	*[%]*	Continuous
81	Blood test: Leukocytes [$\log_{10}$]	*[count/µL]*	Continuous
82	Blood test: Lymphocytes [$\log_{10}$]	*[count/µL]*	Continuous
83	Blood test: Neutrophils [$\log_{10}$]	*[count/µL]*	Continuous
84	Blood test: Monocytes [$\log_{10}$]	*[count/µL]*	Continuous
85	Blood test: Basophils [$\log_{10}$]	*[count/µL]*	Continuous
86	Blood test: Neutrophil to lymphocyte ratio (NLR) [$\log_{10}$]	*[ratio]*	Continuous

Num	Feature	Values or units	Type
87	Blood test: Fibrinogen [$\log_{10}$]	*[mg/dL]*	Continuous
88	Blood test: D-dimer [$\log_{10}$]	*[ng/mL]*	Continuous
89	Blood test: Serum IP-10 [$\log_{10}$]	*[pg/mL]*	Continuous
90	Arterial blood gas tests: $SatO_2$	*[%]*	Continuous
91	Arterial blood gas tests: FiO_2	*[fraction]*	Continuous
92	Arterial blood gas tests: $SatO_2/FiO_2$	*[ratio]*	Continuous
93	COVID-19 diagn: Method, PCR sputum	No, Yes	Binary
94	COVID-19 diagn: Method, PCR nasophar	No, Yes	Binary
95	COVID-19 diagn: Method, Missing	No, Yes	Binary
96	COVID-19 diagn: Days diagnosed before admission	—	Integer
97	COVID-19 diagn: Antigens in urine	No, Yes	Binary
98	Emerg COVID-19 treatm: Antibiotics, Beta-lactam	No, Yes	Binary
99	Emerg COVID-19 treatm: Antibiotics, Macrolides	No, Yes	Binary
100	Emerg COVID-19 treatm: Antibiotics, Macrol & Beta	No, Yes	Binary
101	Emerg COVID-19 treatm: Antibiotics, Quinolones	No, Yes	Binary
102	Emerg COVID-19 treatm: Antibiotics, Others	No, Yes	Binary
103	Emerg COVID-19 treatm: Chloroquine	No, Yes	Binary
104	Emerg COVID-19 treatm: Kaletra	No, Yes	Binary
105	Emerg COVID-19 treatm: Remdesivir	No, Yes	Binary
106	Emerg COVID-19 treatm: Interferon Beta-1a	No, Yes	Binary
107	Emerg COVID-19 treatm: IV corticoids	No = 0, Low = 1, High = 2	Ordinal
108	Emerg COVID-19 treatm: LMWH	No = 0, Prophylaxis = 1, Below therapeutical = 2, Therapeut. = 3, High risk = 4	Ordinal
109	Demographics per postcode: Population density [$\log_{10}$]	*[inhab/km^2]*	Continuous
110	Demographics per postcode: Population under 18 years	*[%]*	Continuous
111	Demographics per postcode: Population over 65 years	*[%]*	Continuous
112	Demographics per postcode: Average age	*[years]*	Continuous
113	Demographics per postcode: Average household size	*[persons/house]*	Continuous
114	Demographics per postcode: Single-person households	*[%]*	Continuous
115	Demographics per postcode: Average personal income	*[k€]*	Continuous
116	Pollut expos: Chronic, 50th perc – PM_{10}	*[μg/m^3]*	Continuous
117	Pollut expos: Chronic, 50th perc – $PM_{2.5}$	*[μg/m^3]*	Continuous
118	Pollut expos: Chronic, 50th perc – O_3	*[μg/m^3]*	Continuous
119	Pollut expos: Chronic, 50th perc – NO_2	*[μg/m^3]*	Continuous
120	Pollut expos: Chronic, 50th perc – NO	*[μg/m^3]*	Continuous
121	Pollut expos: Chronic, 50th perc – NO_X	*[μg/m^3]*	Continuous
122	Pollut expos: Chronic, 50th perc – SO_2	*[μg/m^3]*	Continuous
123	Pollut expos: Chronic, 50th perc – CO	*[mg/m^3]*	Continuous
124	Pollut expos: Chronic, 90th perc – PM_{10}	*[μg/m^3]*	Continuous
125	Pollut expos: Chronic, 90th perc – $PM_{2.5}$	*[μg/m^3]*	Continuous
126	Pollut expos: Chronic, 90th perc – O_3	*[μg/m^3]*	Continuous

Num	Feature	Values or units	Type
127	Pollut expos: Chronic, 90th perc – NO_2	*[μg/m³]*	Continuous
128	Pollut expos: Chronic, 90th perc – NO	*[μg/m³]*	Continuous
129	Pollut expos: Chronic, 90th perc – NO_X	*[μg/m³]*	Continuous
130	Pollut expos: Chronic, 90th perc – SO_2	*[μg/m³]*	Continuous
131	Pollut expos: Chronic, 90th perc – CO	*[mg/m³]*	Continuous
132	Pollut expos: Acute, 50th perc – PM_{10}	*[μg/m³]*	Continuous
133	Pollut expos: Acute, 50th perc – $PM_{2.5}$	*[μg/m³]*	Continuous
134	Pollut expos: Acute, 50th perc – O_3	*[μg/m³]*	Continuous
135	Pollut expos: Acute, 50th perc – NO_2	*[μg/m³]*	Continuous
136	Pollut expos: Acute, 50th perc – NO	*[μg/m³]*	Continuous
137	Pollut expos: Acute, 50th perc – NO_X	*[μg/m³]*	Continuous
138	Pollut expos: Acute, 50th perc – SO_2	*[μg/m³]*	Continuous
139	Pollut expos: Acute, 50th perc – CO	*[mg/m³]*	Continuous
140	Pollut expos: Acute, 90th perc – PM_{10}	*[μg/m³]*	Continuous
141	Pollut expos: Acute, 90th perc – $PM_{2.5}$	*[μg/m³]*	Continuous
142	Pollut expos: Acute, 90th perc – O_3	*[μg/m³]*	Continuous
143	Pollut expos: Acute, 90th perc – NO_2	*[μg/m³]*	Continuous
144	Pollut expos: Acute, 90th perc – NO	*[μg/m³]*	Continuous
145	Pollut expos: Acute, 90th perc – NO_X	*[μg/m³]*	Continuous
146	Pollut expos: Acute, 90th perc – SO_2	*[μg/m³]*	Continuous
147	Pollut expos: Acute, 90th perc – CO	*[mg/m³]*	Continuous

Categorical variables were first transformed via one-hot encoding, having dropped the corresponding default category – *'Comorbidity: Broncho'*=None, *'Emergency treatm: Corticosteroids'*=None, *'Pulmonary status: Infiltr. X-Ray'*=None, *'Pulmonary status: Infiltr. type'*=None, *'COVID-19 diagn: Method'*=Rapid serology, *'Emerg COVID-19 treatm: Antibiotics'*=None.

Abbreviations for Table S1 – *ILD*: interstitial lung disease, *COPD*: chronic obstructive pulmonary disease, *HIV*: human immunodeficiency virus, *PSI*: pneumonia severity index, *CURB-65*: pneumonia severity score (confusion, urea, respiratory rate, blood pressure, age 65), *qSOFA*: quick sequential organ failure assessment score, *ACEI*: angiotensin-converting-enzyme inhibitors, *AIIRB*: angiotensin II receptor blockers, *IP-10*: interferon gamma inducible protein-10, *PCR*: polymerase chain reaction, *IV*: intravenous, *LMWH*: low molecular weight heparin.

S.B Data: Cohort characteristics

Abbreviations for Table S2 – *NA*: not available, *IQR*: inter-quartile range, *K-W*: Kruskal-Wallis, *NS*: not significant, *BMI*: body-mass index, *ILD*: interstitial lung disease, *COPD*: chronic obstructive pulmonary disease, *HIV*: human immunodeficiency virus, *PSI*: pneumonia severity index, *CURB-65*: pneumonia severity score (confusion, urea, respiratory rate, blood pressure, age 65), *qSOFA*: quick sequential organ failure assessment score, *ACEI*: angiotensin-converting-enzyme inhibitors, *AIIRB*: angiotensin II receptor blockers, *BUN*: blood urea nitrogen, *GGT*: gamma-glutamyl transferase, *LDH*: lactate dehydrogenase, *CRP*: C-reactive protein, *PCT*: procalcitonin, *IP-10*: interferon gamma inducible protein-10, *PCR*: polymerase chain reaction, *IV*: intravenous, *LMWH*: low molecular weight heparin.

Table S2. Cohort characteristics. Demographic and clinical characteristics across patients in our cohort: overall, and by severity group.

Variable		**Overall** n=1548	By severity *Low* n=712 (46.0%)	By severity *Medium* n=238 (15.4%)	By severity *High* n=598 (38.6%)	p-value	Effect size	
Hospital	A	358 (23.1%)	205 (57.3%)	36 (10.1%)	117 (32.7%)	χ^2	V	
	B	380 (24.5%)	229 (60.3%)	50 (13.2%)	101 (26.6%)			
	C	438 (28.3%)	119 (27.2%)	59 (13.5%)	260 (59.4%)			
	D	372 (24.0%)	159 (42.7%)	93 (25.0%)	120 (32.3%)	<0.001	0.225	Medium
Sex	Male	952 (61.5%)	385 (40.4%)	143 (15.0%)	424 (44.5%)	χ^2	V	
	Female	596 (38.5%)	327 (54.9%)	95 (15.9%)	174 (29.2%)			
	NA	0	0	0	0	<0.001	0.155	Small
Age [years]	Median	65	60	71	69	K-W	η^2_H	
	IQR	[53, 77]	[49, 72]	[60, 81]	[57, 79]			
	Num. valid	1548 (100%)	712 (100%)	238 (100%)	598 (100%)	<0.001	0.061	Medium
Height [m]	Median	1.66	1.65	1.65	1.68	K-W	η^2_H	
	IQR	[1.60, 1.73]	[1.60, 1.72]	[1.58, 1.73]	[1.60, 1.74]			
	Num. valid	895 (57.8%)	417 (58.6%)	129 (54.2%)	349 (58.4%)	0.016	0.007	Negligible
Weight [kg]	Median	78.5	78.0	75.0	80.0	K-W	η^2_H	
	IQR	[68.0, 89.0]	[66.0, 89.0]	[67.0, 86.0]	[70.0, 90.0]			
	Num. valid	982 (63.4%)	466 (65.4%)	141 (59.2%)	375 (62.7%)	0.009	0.008	Negligible
Body mass index (BMI) [kg/m^2]	Median	27.78	27.76	27.14	28.09	K-W	η^2_H	
	IQR	[25.23, 31.55]	[25.05, 31.74]	[25.07, 30.58]	[25.71, 31.61]			
	Num. valid	866 (55.9%)	403 (56.6%)	126 (52.9%)	337 (56.4%)	0.221	NS	NS
Obesity	No	664 (42.9%)	305 (42.8%)	104 (43.7%)	255 (42.6%)	χ^2	V	
	Yes	343 (22.2%)	162 (22.8%)	44 (18.5%)	137 (22.9%)			
	NA	541 (34.9%)	245 (34.4%)	90 (37.8%)	206 (34.4%)	0.483	NS	NS
Alcohol use	No	941 (60.8%)	432 (60.7%)	166 (69.7%)	343 (57.4%)	χ^2	V	
	Ex	16 (1.0%)	4 (0.6%)	4 (1.7%)	8 (1.3%)			
	Yes	54 (3.5%)	26 (3.7%)	5 (2.1%)	23 (3.8%)			
	NA	537 (34.7%)	250 (35.1%)	63 (26.5%)	224 (37.5%)	0.242	NS	NS
Tobacco use	No	900 (58.1%)	421 (59.1%)	144 (60.5%)	335 (56.0%)	χ^2	V	
	Ex	347 (22.4%)	184 (25.8%)	47 (19.7%)	116 (19.4%)			
	Yes	83 (5.4%)	42 (5.9%)	14 (5.9%)	27 (4.5%)			
	NA	218 (14.1%)	65 (9.1%)	33 (13.9%)	120 (20.1%)	0.346	NS	NS
Lives at nursing home	No	1274 (82.3%)	651 (91.4%)	174 (73.1%)	449 (75.1%)	χ^2	V	
	Yes	94 (6.1%)	15 (2.1%)	37 (15.5%)	42 (7.0%)			
	NA	180 (11.6%)	46 (6.5%)	27 (11.3%)	107 (17.9%)	<0.001	0.209	Small
Comorbidity: Hypertension	No	801 (51.7%)	432 (60.7%)	96 (40.3%)	273 (45.7%)	χ^2	V	
	Yes	474 (48.3%)	280 (39.3%)	142 (59.7%)	325 (54.3%)			
	NA	0	0	0	0	<0.001	0.165	Small
Comorbidity: Diabetes mellitus	No	1218 (78.7%)	582 (81.7%)	188 (79.0%)	448 (74.9%)	χ^2	V	
	Yes	326 (21.1%)	128 (18.0%)	50 (21.0%)	148 (24.7%)			
	NA	4 (0.3%)	2 (0.3%)	0	2 (0.3%)	0.011	0.067	Negligible
Comorbidity: Dyslipidemia	No	973 (62.9%)	475 (66.7%)	128 (53.8%)	370 (61.9%)	χ^2	V	
	Yes	575 (37.1%)	237 (33.3%)	110 (46.2%)	228 (38.1%)			
	NA	0	0	0	0	0.001	0.085	Negligible
Comorbidity: Cardiovascular	No	1264 (81.7%)	613 (86.1%)	197 (82.8%)	454 (75.9%)	χ^2	V	
	Yes	284 (18.3%)	99 (13.9%)	41 (17.2%)	144 (24.1%)			
	NA	0	0	0	0	<0.001	0.116	Small

Variable		Overall<br>n=1548	By severity<br>***Low***<br>n=712 (46.0%)	 ***Medium***<br>n=238 (15.4%)	 ***High***<br>n=598 (38.6%)	p-value	Effect size	
Comorbidity: Chronic heart failure	No	1111 (71.8%)	478 (67.1%)	190 (79.8%)	443 (74.1%)	χ^2	V	
	Yes	78 (5.0%)	29 (4.1%)	12 (5.0%)	37 (6.2%)			
	NA	359 (23.2%)	205 (28.8%)	36 (15.1%)	118 (19.7%)	0.418	NS	NS
Comorbidity: Cerebrovascular	No	1058 (68.3%)	472 (66.3%)	168 (70.6%)	418 (69.9%)	χ^2	V	
	Yes	132 (8.5%)	35 (4.9%)	34 (14.3%)	63 (10.5%)			
	NA	358 (23.1%)	205 (28.8%)	36 (15.1%)	117 (19.6%)	$<$0.001	0.115	Small
Comorbidity: Bronchopathy	No	1232 (79.6%)	559 (78.5%)	203 (85.3%)	470 (78.6%)	χ^2	V	
	Asthma	87 (5.6%)	55 (7.7%)	9 (3.8%)	23 (3.8%)			
	ILD	5 (0.3%)	2 (0.3%)	1 (0.4%)	2 (0.3%)			
	COPD	102 (6.6%)	42 (5.9%)	13 (5.5%)	47 (7.9%)			
	Other	122 (7.9%)	54 (7.6%)	12 (5.0%)	56 (9.4%)			
	NA	0	0	0	0	0.019	0.058	Negligible
Comorbidity: Renal	No	1356 (87.6%)	657 (92.3%)	199 (83.6%)	500 (83.6%)	χ^2	V	
	Yes	186 (12.0%)	55 (7.7%)	38 (16.0%)	93 (15.6%)			
	NA	6 (0.4%)	0	1 (0.4%)	5 (0.8%)	$<$0.001	0.118	Small
Comorbidity: Hepatic	No	1496 (96.6%)	699 (98.2%)	226 (95.0%)	571 (95.5%)	χ^2	V	
	Yes	52 (3.4%)	13 (1.8%)	12 (5.0%)	27 (4.5%)			
	NA	0	0	0	0	0.008	0.007	Negligible
Comorbidity: Ischemia	No	1064 (68.7%)	574 (80.6%)	173 (72.7%)	317 (53.0%)	χ^2	V	
	Yes	46 (3.0%)	19 (2.7%)	6 (2.5%)	21 (3.5%)			
	NA	438 (28.3%)	119 (16.7%)	59 (24.8%)	260 (43.5%)	0.073	NS	NS
Comorbidity: Peptic ulcer	No	1086 (70.2%)	582 (81.7%)	176 (73.9%)	328 (54.8%)	χ^2	V	
	Yes	24 (1.6%)	11 (1.5%)	3 (1.3%)	10 (1.7%)			
	NA	438 (28.3%)	119 (16.7%)	59 (24.8%)	260 (43.5%)	0.478	NS	NS
Comorbidity: Thyroid	No	664 (42.9%)	382 (53.6%)	77 (32.3%)	205 (34.3%)	χ^2	V	
	Yes	73 (4.7%)	52 (7.3%)	9 (3.8%)	12 (2.0%)			
	NA	811 (52.4%)	278 (39.0%)	152 (63.9%)	381 (63.7%)	0.034	0.081	Negligible
Comorbidity: Autoimmune	No	1120 (72.4%)	526 (73.9%)	136 (57.1%)	458 (76.6%)	χ^2	V	
	Yes	52 (3.4%)	26 (3.7%)	9 (3.8%)	17 (2.8%)			
	NA	376 (24.3%)	160 (22.5%)	93 (39.1%)	123 (20.6%)	0.369	NS	NS
Comorbidity: Transplants	No	1094 (70.7%)	588 (82.6%)	174 (73.1%)	332 (55.5%)	χ^2	V	
	Yes	16 (1.0%)	5 (0.7%)	5 (2.1%)	6 (1.0%)			
	NA	438 (28.3%)	119 (16.7%)	59 (24.8%)	260 (43.5%)	0.131	NS	NS
Comorbidity: Cancer, neoplasia	No	1428 (92.2%)	669 (94.0%)	212 (89.1%)	547 (91.5%)	χ^2	V	
	Yes	120 (7.8%)	43 (6.0%)	26 (10.9%)	51 (8.5%)			
	NA	0	0	0	0	0.034	0.056	Negligible
Comorbidity: Immune, HIV	No	1431 (92.4%)	692 (97.2%)	214 (89.9%)	525 (87.8%)	χ^2	V	
	Yes	67 (4.3%)	12 (1.7%)	20 (8.4%)	35 (5.9%)			
	NA	50 (3.2%)	8 (1.1%)	4 (1.7%)	38 (6.4%)	$<$0.001	0.126	Small
Comorbidity: Overall	No	381 (24.6%)	228 (32.0%)	41 (17.2%)	112 (18.7%)	χ^2	V	
	Yes	1167 (75.4%)	484 (68.0%)	197 (82.8%)	486 (81.3%)			
	NA	0	0	0	0	$<$0.001	0.155	Small
Comorbidity: Charlson index	Median	3	2	4	4			
	IQR	[1, 5]	[1, 4]	[2, 6]	[2, 6]	K-W	η^2_H	
	Num. valid	1548 (100%)	712 (100%)	238 (100%)	598 (100%)	$<$0.001	0.071	Medium
Pneumonia: PSI score	Median	70	59	78	83	K-W	η^2_H	
	IQR	[53, 92]	[46, 77]	[62, 100]	[65, 109]			

Variable		Overall	By severity Low	Medium	High	p-value	Effect size	
		n=1548	n=712 (46.0%)	n=238 (15.4%)	n=598 (38.6%)			
	Num. valid	1287 (83.1%)	640 (89.9%)	200 (84.0%)	447 (74.7%)	<0.001	0.158	Large
Pneumonia: CURB-65 score	0	539 (34.8%)	337 (47.3%)	59 (24.8%)	143 (23.9%)	χ^2	V	
	1	516 (33.3%)	230 (32.3%)	80 (33.6%)	206 (34.4%)			
	2	361 (23.3%)	125 (17.6%)	68 (28.6%)	168 (28.1%)			
	3	89 (5.7%)	10 (1.4%)	20 (8.4%)	59 (9.9%)			
	4	20 (1.3%)	3 (0.4%)	3 (1.3%)	14 (2.3%)			
	NA	23 (1.5%)	7 (30.4%)	8 (3.4%)	8 (1.3%)	<0.001	0.201	Small
Sepsis: qSOFA score	0	791 (51.1%)	502 (70.5%)	131 (55.0%)	158 (26.4%)	χ^2	V	
	1	274 (17.7%)	84 (11.8%)	41 (17.2%)	149 (24.9%)			
	2	39 (2.5%)	6 (0.8%)	6 (2.5%)	27 (4.5%)			
	3	3 (0.2%)	1 (0.1%)	0	2 (0.3%)			
	NA	441 (28.5%)	119 (16.7%)	60 (13.6%)	262 (43.8%)	<0.001	0.259	Medium
Symptoms: Cough	No	404 (26.1%)	193 (27.1%)	72 (30.3%)	139 (23.2%)	χ^2	V	
	Yes	1015 (65.6%)	488 (68.5%)	144 (60.5%)	383 (64.0%)			
	NA	129 (8.3%)	31 (4.4%)	22 (9.2%)	76 (12.7%)	0.184	NS	NS
Symptoms: Expectoration	No	1166 (75.3%)	567 (79.6%)	181 (76.1%)	418 (69.9%)	χ^2	V	
	Yes	254 (16.4%)	114 (16.0%)	35 (14.7%)	105 (17.6%)			
	NA	128 (8.3%)	31 (4.4%)	22 (9.2%)	75 (12.5%)	0.255	NS	NS
Symptoms: Dyspnea	No	754 (48.7%)	387 (54.4%)	133 (55.9%)	234 (39.1%)	χ^2	V	
	Yes	669 (43.2%)	296 (41.6%)	83 (34.9%)	290 (48.5%)			
	NA	125 (8.1%)	29 (4.1%)	22 (9.2%)	74 (12.4%)	<0.001	0.126	Small
Symptoms: Myalgia	No	1108 (71.6%)	519 (72.9%)	165 (69.3%)	424 (70.9%)	χ^2	V	
	Yes	314 (20.3%)	164 (23.0%)	51 (21.4%)	99 (16.6%)			
	NA	126 (8.1%)	29 (4.1%)	22 (9.2%)	75 (12.5%)	0.091	NS	NS
Symptoms: Confusion	No	1336 (86.3%)	672 (94.4%)	198 (83.2%)	466 (77.9%)	χ^2	V	
	Yes	86 (5.6%)	11 (1.5%)	19 (8.0%)	56 (9.4%)			
	NA	126 (9.1%)	29 (4.1%)	21 (8.8%)	76 (12.7%)	<0.001	0.177	Small
Symptoms: Thorax pain	No	1279 (82.6%)	614 (86.2%)	190 (79.8%)	475 (79.4%)	χ^2	V	
	Yes	143 (9.2%)	69 (9.7%)	26 (10.9%)	48 (8.0%)			
	NA	126 (8.1%)	29 (4.1%)	22 (9.2%)	75 (12.5%)	0.500	NS	NS
Symptoms: Anosmia	No	1320 (85.3%)	572 (80.3%)	212 (89.1%)	536 (89.6%)	χ^2	V	
	Yes	219 (14.1%)	137 (19.2%)	26 (10.9%)	56 (9.4%)			
	NA	9 (0.6%)	3 (0.4%)	0	6 (1.0%)	<0.001	0.131	Small
Symptoms: Fever	No	235 (15.2%)	110 (15.4%)	44 (18.5%)	81 (13.5%)	χ^2	V	
	Febricula	272 (17.6%)	162 (22.8%)	32 (13.4%)	78 (13.0%)			
	Fever	916 (59.2%)	411 (57.7%)	140 (58.8%)	365 (61.0%)			
	NA	125 (8.1%)	29 (4.1%)	22 (9.2%)	74 (12.4%)	<0.001	0.078	Small
Symptoms: Digestive-gastro	No	1053 (68.0%)	493 (69.2%)	152 (63.9%)	408 (68.2%)	χ^2	V	
	Yes	353 (22.8%)	179 (25.1%)	62 (26.1%)	112 (18.7%)			
	NA	142 (9.2%)	40 (5.6%)	24 (10.1%)	78 (13.0%)	0.048	0.054	Negligible
Symptoms: Overall	No	1 (0.1%)	0	0	1 (0.2%)	χ^2	V	
	Yes	1547 (99.9%)	712 (100%)	238 (100%)	597 (99.8%)			
	NA	0	0	0	0	0.452	NS	NS
Symptoms: Days	Median	7	7	6	6	K-W	η^2_H	
	IQR	[4, 10]	[5, 10]	[4, 9]	[4, 8]			
	Num. valid	1415 (91.4%)	682 (95.8%)	213 (89.5%)	520 (87.0%)	<0.001	0.030	Small
Emergency treatm: ACEI-AIIRB	No	745 (45.1%)	426 (59.8%)	113 (47.5%)	206 (34.4%)	χ^2	V	

Variable		**Overall**	**By severity** *Low*	*Medium*	*High*			
		n=1548	n=712 (46.0%)	n=238 (15.4%)	n=598 (38.6%)	p-value	Effect size	
	Yes	365 (23.6%)	167 (23.5%)	66 (27.7%)	132 (22.1%)			
	NA	438 (28.3%)	119 (16.7%)	59 (24.8%)	260 (43.5%)	0.001	0.100	Small
Emergency treatm: Statin	No	960 (62.0%)	496 (69.7%)	133 (55.9%)	331 (55.4%)	χ^2	V	
	Yes	410 (26.5%)	172 (24.2%)	78 (32.8%)	160 (26.8%)			
	NA	178 (11.5%)	44 (6.2%)	27 (11.3%)	107 (17.9%)	0.002	0.086	Negligible
Emergency treatm: Anticoagulant	No	1406 (90.8%)	666 (93.5%)	209 (87.8%)	531 (88.8%)	χ^2	V	
	Yes	142 (9.2%)	46 (6.5%)	29 (12.2%)	67 (11.2%)			
	NA	0	0	0	0	0.003	0.080	Negligible
Emergency treatm: Antiplatelet	No	980 (63.3%)	525 (73.7%)	162 (68.1%)	293 (49.0%)	χ^2	V	
	Yes	165 (10.7%)	73 (10.3%)	26 (10.9%)	66 (11.0%)			
	NA	403 (26.0%)	114 (16.0%)	50 (21.0%)	239 (40.0%)	0.030	0.066	Negligible
Emergency treatm: Corticosteroids	No	1180 (76.2%)	567 (79.6%)	187 (78.6%)	426 (71.2%)	χ^2	V	
	Inhaled	145 (9.4%)	86 (12.1%)	12 (5.0%)	47 (7.9%)			
	Oral	44 (2.8%)	14 (2.0%)	12 (5.0%)	18 (3.0%)			
	NA	179 (11.6%)	45 (6.3%)	27 (11.3%)	107 (17.9%)	0.003	0.066	Negligible
Admission status: Body temperature [°C]	Median	37.0	37.0	36.8	37.2	K-W	η^2_H	
	IQR	[36.4, 37.7]	[36.3, 37.6]	[36.4, 37.5]	[36.5, 38.0]			
	Num. valid	1347 (87.0%)	667 (93.7%)	208 (87.4%)	472 (78.9%)	<0.001	0.015	Small
Admission status: Systolic blood pressure [mmHg]	Median	127	127	133	124	K-W	η^2_H	
	IQR	[115, 142]	[115, 139]	[117, 149]	[113, 142]			
	Num. valid	1339 (86.5%)	665 (93.4%)	208 (87.4%)	466 (77.9%)	0.003	0.007	Negligible
Admission status: Diastolic blood pressure [mmHg]	Median	75	77	75	74	K-W	η^2_H	
	IQR	[68, 83]	[70, 85]	[66, 83]	[66, 81]			
	Num. valid	1338 (86.4%)	665 (93.4%)	208 (87.4%)	465 (77.8%)	<0.001	0.019	Small
Admission status: Respiratory rate [min^{-1}]	Median	18	17	18	24	K-W	η^2_H	
	IQR	[16, 24]	[16, 20]	[16, 20]	[18, 30]			
	Num. valid	1021 (66.0%)	468 (65.7%)	175 (73.5%)	378 (63.2%)	<0.001	0.173	Large
Admission status: Heart rate [min^{-1}]	Median	90	92	88	90	K-W	η^2_H	
	IQR	[80, 102]	[80, 103]	[76, 102]	[80, 102]			
	Num. valid	1525 (98.5%)	710 (99.7%)	237 (99.6%)	578 (96.7%)	0.035	0.003	Negligible
Admission status: SpO_2 [%]	Median	95	96	95	94	K-W	η^2_H	
	IQR	[93, 97]	[94, 97]	[93, 97]	[90, 96]			
	Num. valid	1529 (98.8%)	709 (99.6%)	236 (99.2%)	584 (97.7%)	<0.001	0.080	Medium
Admission status: FiO_2 [fraction]	Median	0.21	0.21	0.21	0.21	K-W	η^2_H	
	IQR	[0.21, 0.21]	[0.21, 0.21]	[0.21, 0.21]	[0.21, 0.21]			
	Num. valid	1526 (98.6%)	710 (99.7%)	236 (99.2%)	580 (97.0%)	<0.001	0.063	Medium
Admission status: SpO_2/FiO_2 [ratio]	Median	452.38	457.14	452.38	438.09	K-W	η^2_H	
	IQR	[433.33, 461.90]	[447.62, 461.90]	[442.86, 461.90]	[390.48, 452.38]			
	Num. valid	1520 (98.2%)	707 (99.3%)	235 (98.7%)	578 (96.7%)	<0.001	0.139	Medium
Admission status: $SpO_2/RespRate$ [%/min^{-1}]	Median	5.17	5.56	5.28	4.08	K-W	η^2_H	
	IQR	[4.00, 5.94]	[4.83, 6.06]	[4.70, 5.94]	[3.07, 5.22]			
	Num. valid	1017 (65.7%)	468 (65.7%)	175 (73.5%)	374 (62.5%)	<0.001	0.198	Large
Pulmonary status: Crackles	No	390 (25.2%)	246 (34.6%)	45 (18.9%)	99 (16.6%)	χ^2	V	
	Yes	601 (38.8%)	261 (36.7%)	70 (29.4%)	270 (45.2%)			
	NA	557 (36.0%)	205 (28.8%)	123 (51.7%)	229 (38.3%)	<0.001	0.201	Small
Pulmonary status: Infiltr. X-Ray	No	116 (7.5%)	73 (10.3%)	22 (9.2%)	21 (3.5%)	χ^2	V	
	Unilobar	227 (14.7%)	142 (19.9%)	36 (15.1%)	49 (8.2%)			
	Multilob unilat	65 (4.2%)	44 (6.2%)	12 (5.0%)	9 (1.5%)			

Variable		Overall	By severity Low	Medium	High	p-value	Effect size	
		n=1548	n=712 (46.0%)	n=238 (15.4%)	n=598 (38.6%)			
	Bilateral	701 (45.3%)	334 (46.9%)	109 (45.8%)	258 (43.1%)			
	NA	439 (28.4%)	119 (16.7%)	59 (24.8%)	261 (43.6%)	<0.001	0.124	Small
Pulmonary status: Infiltr. type	No	117 (7.6%)	74 (10.4%)	22 (9.2%)	21 (3.5%)	χ^2	V	
	Alveolar	334 (21.6%)	174 (24.4%)	47 (19.7%)	113 (18.9%)			
	Interstitial	770 (49.7%)	316 (44.4%)	125 (52.5%)	329 (55.0%)			
	Consolidation	163 (10.5%)	102 (14.3%)	21 (8.8%)	40 (6.7%)			
	NA	164 (10.6%)	46 (6.5%)	23 (9.7%)	95 (15.9%)	<0.001	0.127	Small
Pulmonary status: Infiltration, Num. lobes	0	123 (7.9%)	75 (10.5%)	22 (9.2%)	26 (4.4%)	χ^2	V	
	1	253 (16.3%)	149 (20.9%)	40 (16.8%)	64 (10.7%)			
	2	406 (26.2%)	197 (27.7%)	68 (28.6%)	141 (23.6%)			
	3	205 (13.2%)	105 (14.7%)	35 (14.7%)	65 (10.9%)			
	4	188 (12.1%)	91 (12.8%)	22 (9.2%)	75 (12.5%)			
	5	119 (7.7%)	36 (5.1%)	17 (7.1%)	66 (11.0%)			
	6	49 (3.2%)	12 (1.7%)	2 (0.9%)	35 (5.8%)			
	NA	205 (13.2%)	47 (6.6%)	32 (13.4%)	126 (21.1%)	<0.001	0.157	Small
Pulmonary status: Pleural effusion	No	1317 (85.1%)	652 (91.6%)	200 (84.0%)	465 (77.8%)	χ^2	V	
	Yes	37 (2.4%)	13 (1.8%)	7 (2.9%)	17 (2.8%)			
	NA	194 (12.5%)	47 (6.6%)	31 (13.0%)	116 (19.4%)	0.225	NS	NS
Blood test: Glucose [mg/dL]	Median	111	107	113	117	K-W	η^2_H	
	IQR	[99, 134]	[97, 125]	[100, 143]	[103, 142]			
	Num. valid	1383 (89.3%)	668 (93.8%)	217 (91.2%)	498 (83.3%)	<0.001	0.029	Small
Blood test: Urea [mg/dL]	Median	34	30	39	41	K-W	η^2_H	
	IQR	[26, 47]	[23, 40]	[30, 57]	[30, 61]			
	Num. valid	1107 (71.5%)	592 (83.1%)	178 (74.8%)	337 (56.4%)	<0.001	0.099	Medium
Blood test: Creatinine [mg/dL]	Median	0.92	0.85	0.97	1.00	K-W	η^2_H	
	IQR	[0.75, 1.13]	[0.70, 1.01]	[0.80, 1.24]	[0.81, 1.32]			
	Num. valid	1476 (95.3%)	692 (97.2%)	229 (96.2%)	555 (92.8%)	<0.001	0.067	Medium
Blood test: Blood urea nitrogen (BUN) [mg/dL]	Median	17.0	15.0	20.3	20.0	K-W	η^2_H	
	IQR	[13.0, 23.0]	[12.0, 20.0]	[15.0, 27.0]	[15.0, 28.5]			
	Num. valid	736 (47.5%)	433 (60.8%)	86 (36.1%)	217 (36.3%)	<0.001	0.087	Medium
Blood: Sodium [mEq/L]	Median	138	138	138	137	K-W	η^2_H	
	IQR	[136, 140]	[136, 140]	[136, 141]	[135, 140]			
	Num. valid	1396 (90.2%)	673 (94.5%)	218 (91.6%)	505 (84.4%)	<0.001	0.009	Negligible
Blood test: Gamma-glutamyl transferase (GGT) [U/L]	Median	27	26	23	29	K-W	η^2_H	
	IQR	[18, 44]	[18, 44]	[15, 39]	[19, 47]			
	Num. valid	1265 (81.7%)	639 (89.7%)	197 (82.8%)	429 (71.7%)	0.001	0.009	Negligible
Blood test: Lactate dehydrogenase (LDH) [U/L]	Median	301.5	272.0	283.0	375.0	K-W	η^2_H	
	IQR	[238.0, 389.8]	[224.5, 341.5]	[230.0, 347.8]	[278.0, 470.0]			
	Num. valid	1162 (75.1%)	555 (77.9%)	186 (78.2%)	421 (70.4%)	<0.001	0.122	Medium
Blood test: C-reactive protein (CRP) [mg/L]	Median	72.13	52.06	58.46	108.30	K-W	η^2_H	
	IQR	[32.30, 134.04]	[23.86, 102.25]	[26.64, 119.36]	[60.15, 186.70]			
	Num. valid	1473 (95.2%)	690 (96.9%)	230 (96.6%)	553 (92.5%)	<0.001	0.116	Medium
Blood test: Procalcitonin (PCT) [µg/L]	Median	0.11	0.08	0.09	0.17	K-W	η^2_H	
	IQR	[0.06, 0.22]	[0.05, 0.14]	[0.06, 0.16]	[0.10, 0.37]			
	Num. valid	1089 (70.3%)	485 (68.1%)	172 (72.3%)	432 (72.2%)	<0.001	0.134	Medium
Blood test: Hematocrit [%]	Median	41.0	41.3	40.3	40.9	K-W	η^2_H	
	IQR	[37.7, 44.1]	[38.6, 44.0]	[36.8, 44.6]	[37.1, 44.2]			
	Num. valid	1371 (88.6%)	663 (93.1%)	215 (90.3%)	493 (82.4%)	0.037	0.003	Negligible

Variable		**Overall** n=1548	**By severity** ***Low*** n=712 (46.0%)	***Medium*** n=238 (15.4%)	***High*** n=598 (38.6%)	p-value	Effect size	
Blood test: Leukocytes [count/µL]	Median	6240	6100	6170	6500	K-W	η^2_H	
	IQR	[4710, 8373]	[4705, 7900]	[4378, 8393]	[4900, 9255]			
	Num. valid	1460 (94.3%)	687 (96.5%)	226 (95.0%)	547 (91.5%)	0.013	0.005	Negligible
Blood test: Lymphocytes [count/µL]	Median	900	1000	900	730	K-W	η^2_H	
	IQR	[640, 1240]	[770, 1330]	[650, 1260]	[500, 1070]			
	Num. valid	1530 (98.8%)	706 (99.2%)	235 (98.7%)	589 (98.5%)	<0.001	0.075	Medium
Blood test: Neutrophils [count/µL]	Median	4700	4345	4630	5100	K-W	η^2_H	
	IQR	[3300, 6620]	[3200, 6040]	[3050, 6530]	[3570, 7600]			
	Num. valid	1545 (99.8%)	710 (99.7%)	238 (100%)	597 (99.8%)	<0.001	0.020	Small
Blood test: Monocytes [count/µL]	Median	420	430	480	400	K-W	η^2_H	
	IQR	[290, 590]	[310, 580]	[315, 665]	[270, 560]			
	Num. valid	1108 (71.6%)	592 (83.1%)	179 (75.2%)	337 (56.4%)	0.001	0.011	Small
Blood test: Basophils [count/µL]	Median	20	20	20	10	K-W	η^2_H	
	IQR	[10, 20]	[10, 20]	[10, 20]	[10, 20]			
	Num. valid	684 (44.2%)	364 (51.1%)	127 (53.4%)	193 (32.3%)	0.034	0.007	Negligible
Blood test: Neutrophil to lymphocyte ratio (NLR) [ratio]	Median	4.98	4.17	4.76	7.17	K-W	η^2_H	
	IQR	[3.33, 8.61]	[2.92, 6.33]	[3.43, 4.76]	[4.19, 11.9]			
	Num. valid	1529 (98.8%)	705 (99.0%)	235 (98.7%)	589 (98.5%)	<0.001	0.092	Medium
Blood test: Fibrinogen [mg/dL]	Median	700	685	700	700	K-W	η^2_H	
	IQR	[562, 744]	[531, 716]	[579, 702]	[650, 750]			
	Num. valid	654 (42.2%)	360 (50.6%)	107 (45.0%)	187 (31.3%)	<0.001	0.023	Small
Blood test: D-dimer [ng/mL]	Median	751	610	728	1000	K-W	η^2_H	
	IQR	[430, 1340]	[385, 1010]	[436, 1350]	[570, 1919]			
	Num. valid	1268 (81.9%)	625 (87.8%)	195 (81.9%)	448 (74.9%)	<0.001	0.059	Small
Blood test: Serum IP-10 [pg/mL]	Median	93	97	89	89	K-W	η^2_H	
	IQR	[81, 100]	[85, 100]	[82, 100]	[72, 100]			
	Num. valid	662 (42.8%)	385 (54.1%)	79 (33.2%)	198 (33.1%)	<0.001	0.032	Small
Arterial blood gas test: $SatO_2$ [%]	Median	95	96	95	94	K-W	η^2_H	
	IQR	[93, 97]	[94, 97]	[93, 97]	[90, 96]			
	Num. valid	743 (48.0%)	386 (54.2%)	81 (34.0%)	276 (46.2%)	<0.001	0.093	Medium
Arterial blood gas test: FiO_2 [fraction]	Median	0.21	0.21	0.21	0.21	K-W	η^2_H	
	IQR	[0.21, 0.21]	[0.21, 0.21]	[0.21, 0.21]	[0.21, 0.28]			
	Num. valid	946 (61.1%)	439 (61.7%)	132 (55.5%)	375 (62.7%)	<0.001	0.107	Medium
Arterial blood gas test: $SatO_2/FiO_2$ [ratio]	Median	452.38	457.14	452.38	428.57	K-W	η^2_H	
	IQR	[420.24, 461.90]	[447.62, 461.90]	[439.52, 461.90]	[317.59, 452.38]			
	Num. valid	730 (47.2%)	382 (53.7%)	81 (34.0%)	267 (44.6%)	<0.001	0.163	Large
COVID-19 diagn: Method	Rapid serology	50 (3.2%)	33 (4.6%)	11 (4.6%)	6 (1.0%)	χ^2	V	
	PCR sputum	27 (1.7%)	12 (1.7%)	5 (2.1%)	10 (1.7%)			
	PCR nasophar	880 (56.8%)	464 (65.2%)	139 (58.4%)	277 (46.3%)			
	NA	591 (38.2%)	203 (28.5%)	83 (34.9%)	305 (51.0%)	0.051	NS	NS
COVID-19 diagn: Days diagnosed before admission	Median	0	1	1	0	K-W	η^2_H	
	IQR	[0, 1]	[0, 1]	[0, 1]	[0, 1]			
	Num. valid	811 (52.4%)	444 (62.4%)	131 (55.0%)	236 (39.5%)	0.241	NS	NS
COVID-19 diagn: Antigens in urine	No	662 (42.8%)	319 (44.8%)	98 (41.2%)	245 (41.0%)	χ^2	V	
	Yes	42 (2.7%)	17 (2.4%)	8 (3.4%)	17 (2.8%)			
	NA	844 (54.5%)	376 (52.8%)	132 (55.5%)	336 (56.2%)	0.579	NS	NS
Emerg COVID-19 treatm: Antibiotics	No	682 (44.1%)	301 (42.3%)	78 (32.8%)	303 (50.7%)	χ^2	V	
	Beta-lactam	230 (14.9%)	128 (18.0%)	37 (15.5%)	65 (10.9%)			

Variable		**Overall** n=1548	**By severity** *Low* n=712 (46.0%)	*Medium* n=238 (15.4%)	*High* n=598 (38.6%)	p-value	Effect size	
	Macrolides	89 (5.8%)	55 (7.7%)	16 (6.7%)	18 (3.0%)			
	Macrol & Beta	486 (31.4%)	209 (29.4%)	99 (41.6%)	178 (29.8%)			
	Quinolones	30 (1.9%)	17 (2.4%)	2 (0.8%)	11 (1.8%)			
	Others	31 (2.0%)	2 (0.3%)	6 (2.5%)	23 (3.8%)			
	NA	0	0	0	0	<0.001	0.139	Small
Emerg COVID-19 treatm: Chloroquine	No	485 (31.3%)	240 (33.7%)	57 (23.9%)	188 (31.4%)	χ^2	V	
	Yes	1063 (68.7%)	472 (66.3%)	181 (76.1%)	410 (68.6%)			
	NA	0	0	0	0	0.019	0.062	Negligible
Emerg COVID-19 treatm: Kaletra	No	814 (52.6%)	404 (56.7%)	130 (54.6%)	280 (46.8%)	χ^2	V	
	Yes	734 (47.4%)	308 (43.3%)	108 (45.4%)	318 (53.2%)			
	NA	0	0	0	0	0.001	0.085	Negligible
Emerg COVID-19 treatm: Remdesivir	No	1537 (99.3%)	708 (99.4%)	235 (98.7%)	594 (99.3%)	χ^2	V	
	Yes	11 (0.7%)	4 (0.6%)	3 (1.3%)	4 (0.7%)			
	NA	0	0	0	0	0.533	NS	NS
Emerg COVID-19 treatm: Interferon Beta-1a	No	1436 (92.8%)	703 (98.7%)	229 (96.2%)	504 (84.3%)	χ^2	V	
	Yes	112 (7.2%)	9 (1.3%)	9 (3.8%)	94 (15.7%)			
	NA	0	0	0	0	<0.001	0.260	Small
Emerg COVID-19 treatm: IV corticoids	No	1162 (75.1%)	596 (83.7%)	191 (80.3%)	375 (62.7%)	χ^2	V	
	Low	43 (2.8%)	24 (3.4%)	6 (2.5%)	13 (2.2%)			
	High	105 (6.8%)	33 (4.6%)	8 (3.4%)	64 (10.7%)			
	NA	238 (15.4%)	59 (8.3%)	33 (13.9%)	146 (24.4%)	<0.001	0.111	Small
Emerg COVID-19 treatm: LMWH	No	208 (13.4%)	122 (17.1%)	24 (10.1%)	62 (10.4%)	χ^2	V	
	Prophylaxis	682 (44.1%)	403 (56.6%)	119 (50.0%)	160 (26.8%)			
	Below therap.	67 (4.3%)	16 (2.2%)	10 (4.2%)	41 (6.9%)			
	Therapeut.	94 (6.1%)	28 (3.9%)	23 (9.7%)	43 (7.2%)			
	High risk	58 (3.7%)	24 (3.4%)	3 (1.3%)	31 (5.2%)			
	NA	439 (28.4%)	119 (16.7%)	59 (24.8%)	261 (43.6%)	<0.001	0.191	Small

Univariate statistical comparisons for discrete variables were performed by means of the χ^2 test, and bias-corrected Cramer's V effect size [1].
For continuous variables, univariate comparisons were made with the non-parametric Kruskal-Wallis test, and its corresponding η^2_H effect size.
Thresholds for interpreting effect sizes were taken from [2].

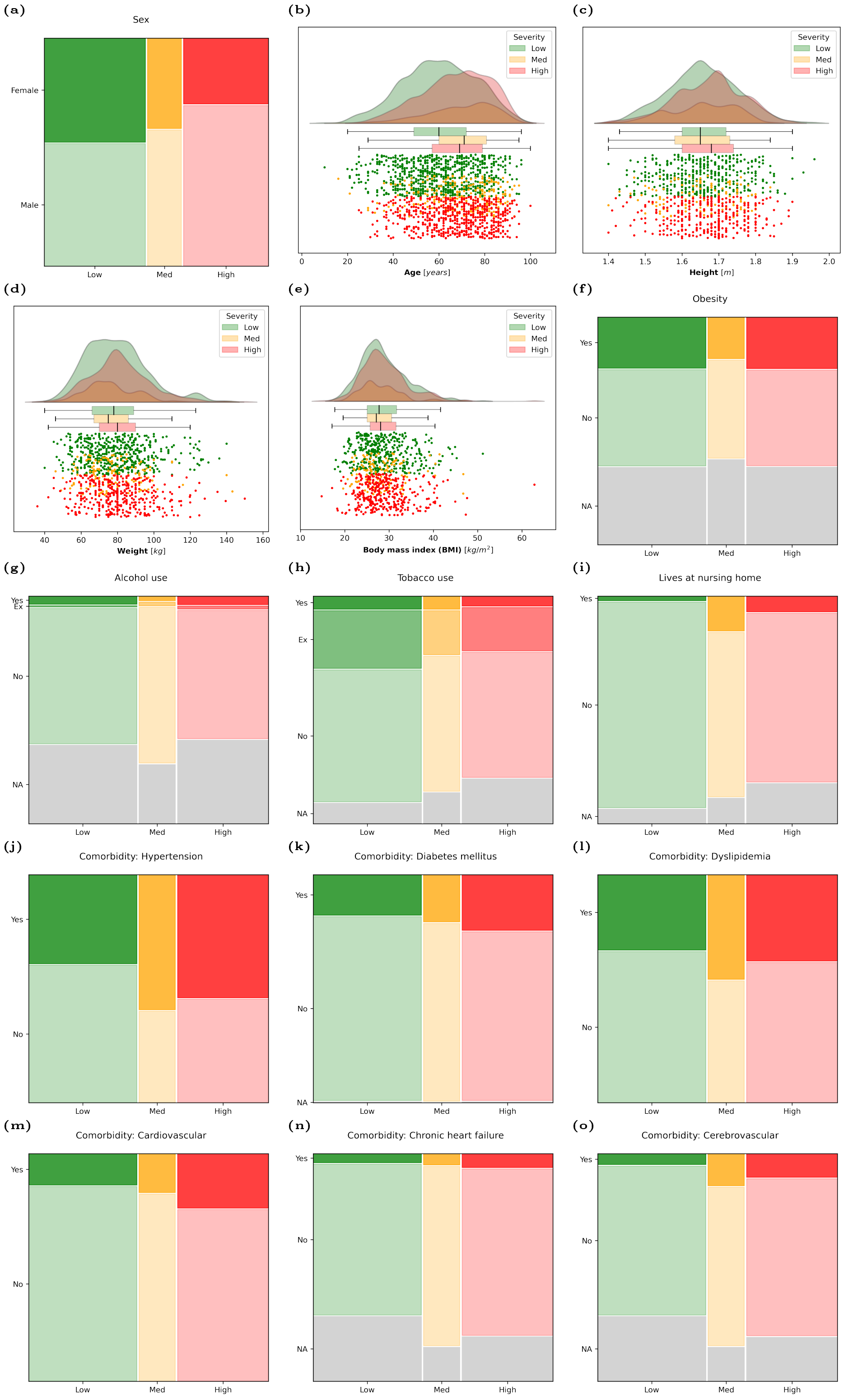

Fig S1. Cohort characteristics (1 of 7). Univariate distributions, by severity.

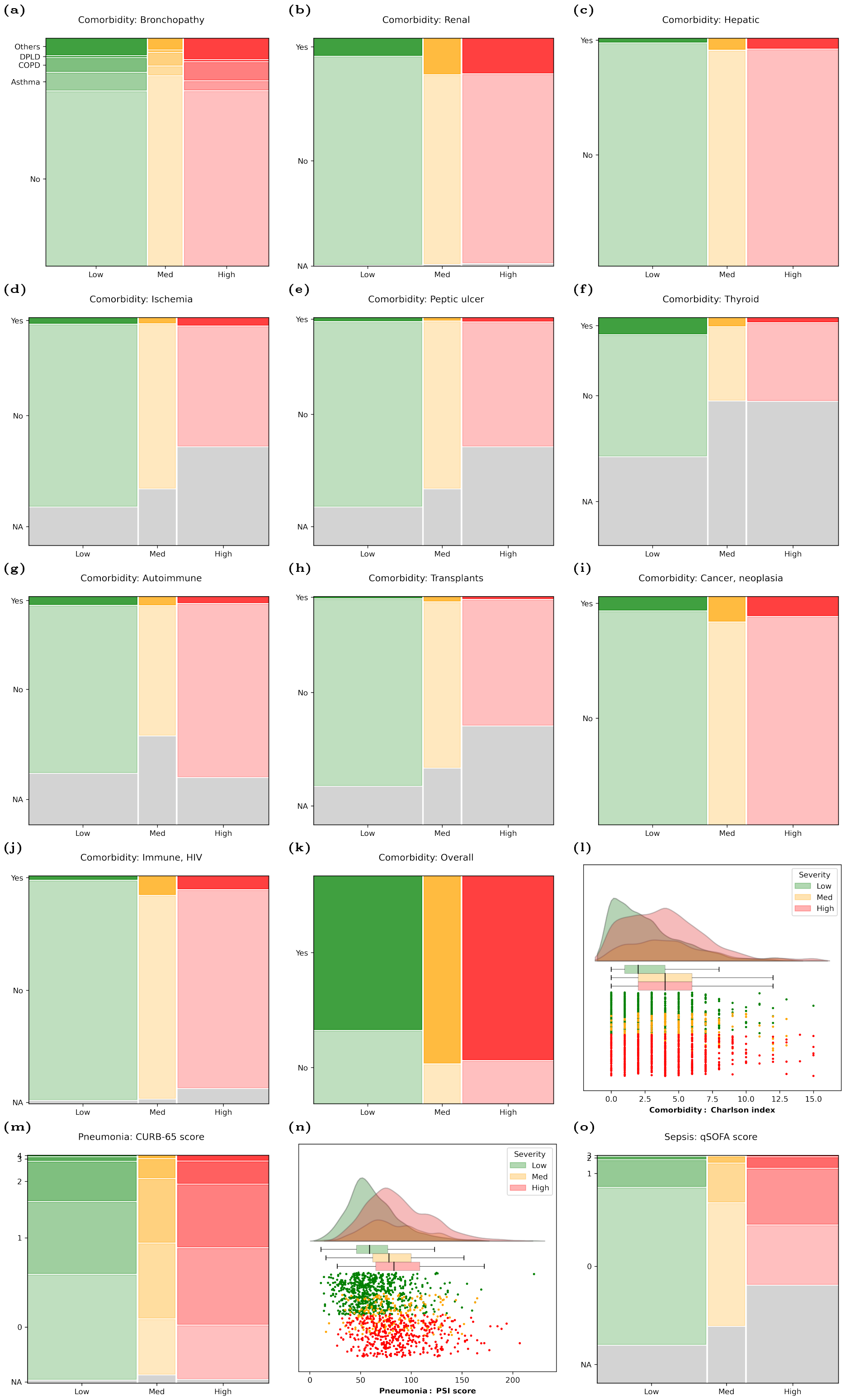

Fig S2. Cohort characteristics (2 of 7). Univariate distributions, by severity.

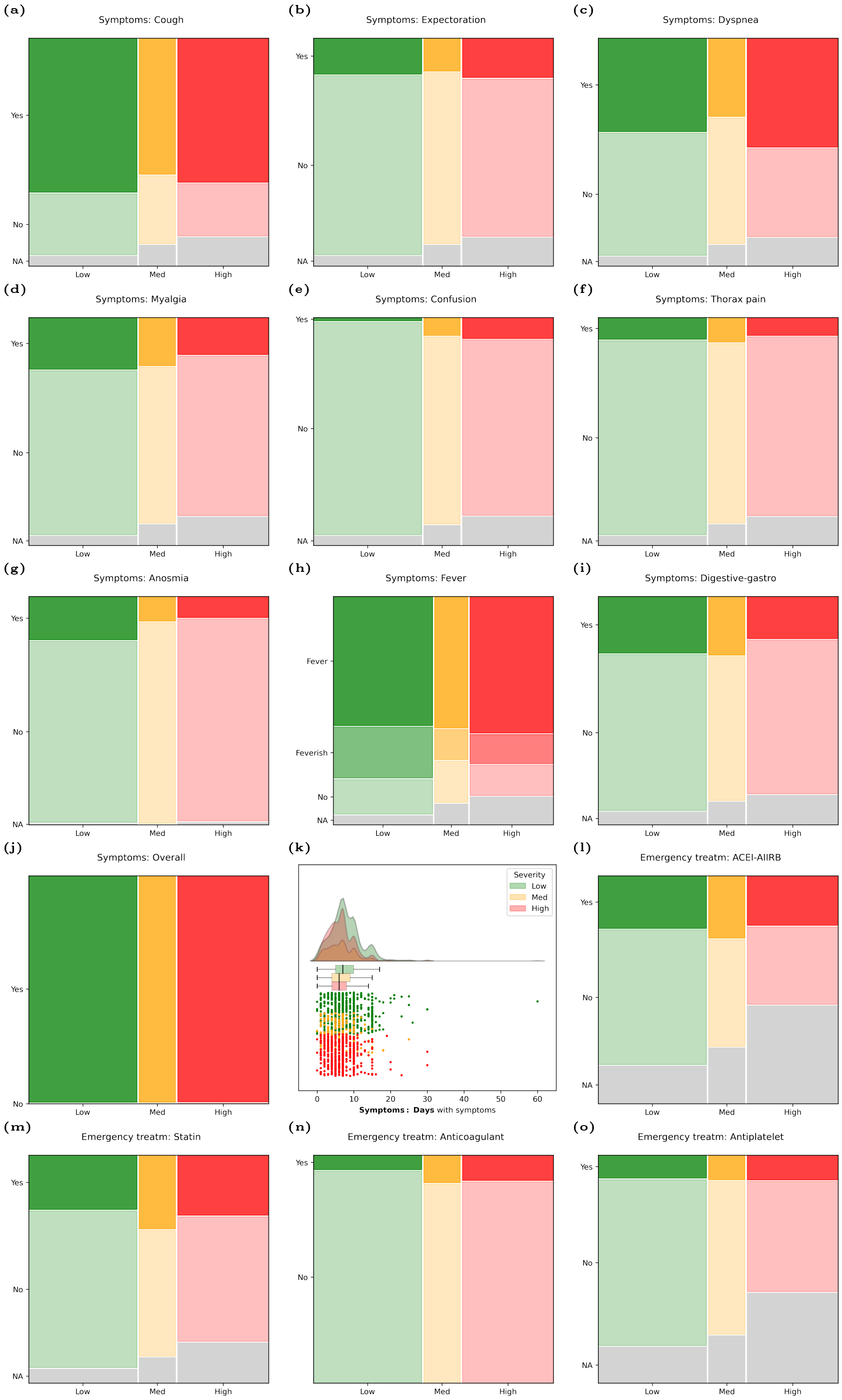

Fig S3. Cohort characteristics (3 of 7). Univariate distributions, by severity.

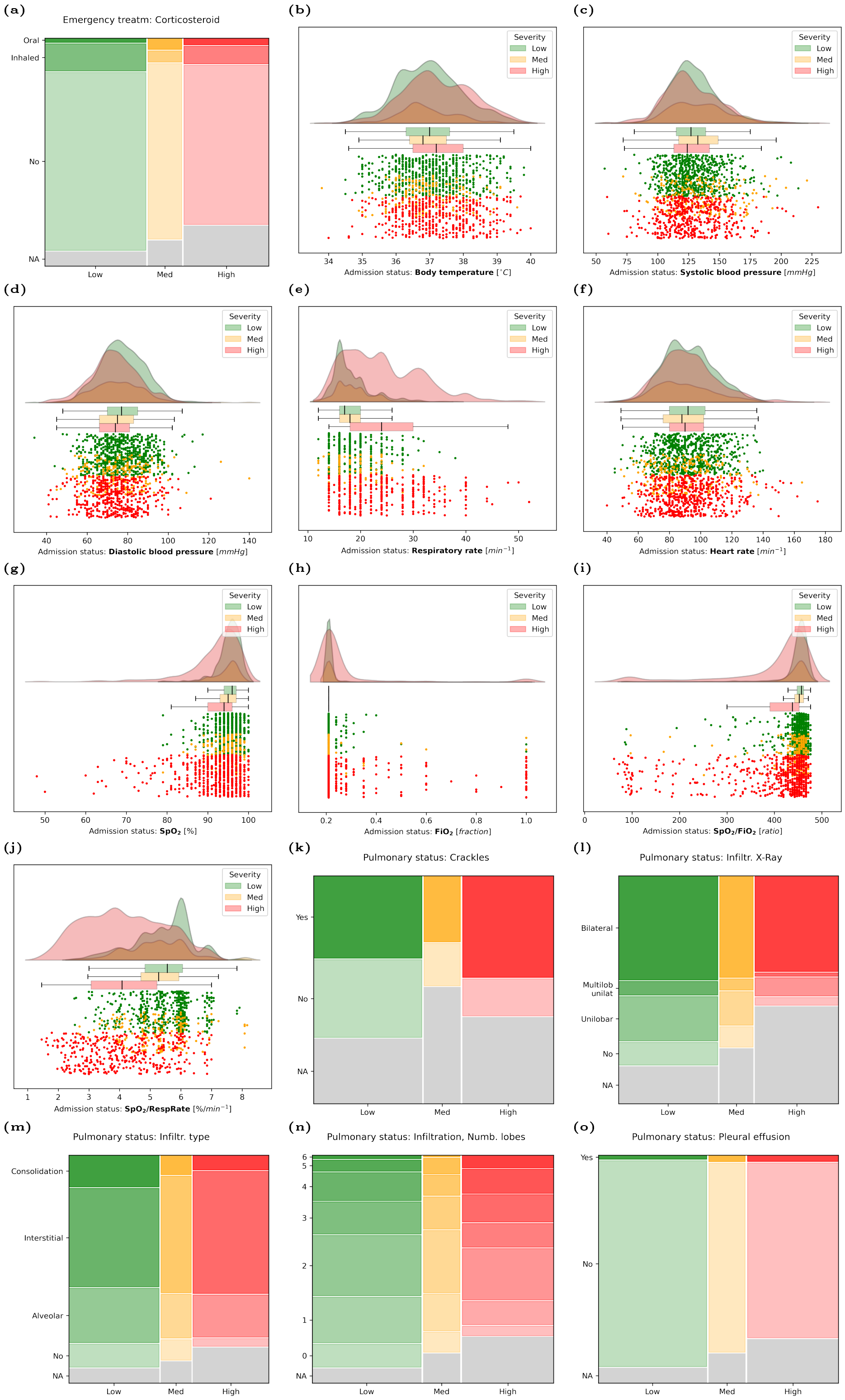

Fig S4. Cohort characteristics (4 of 7). Univariate distributions, by severity.

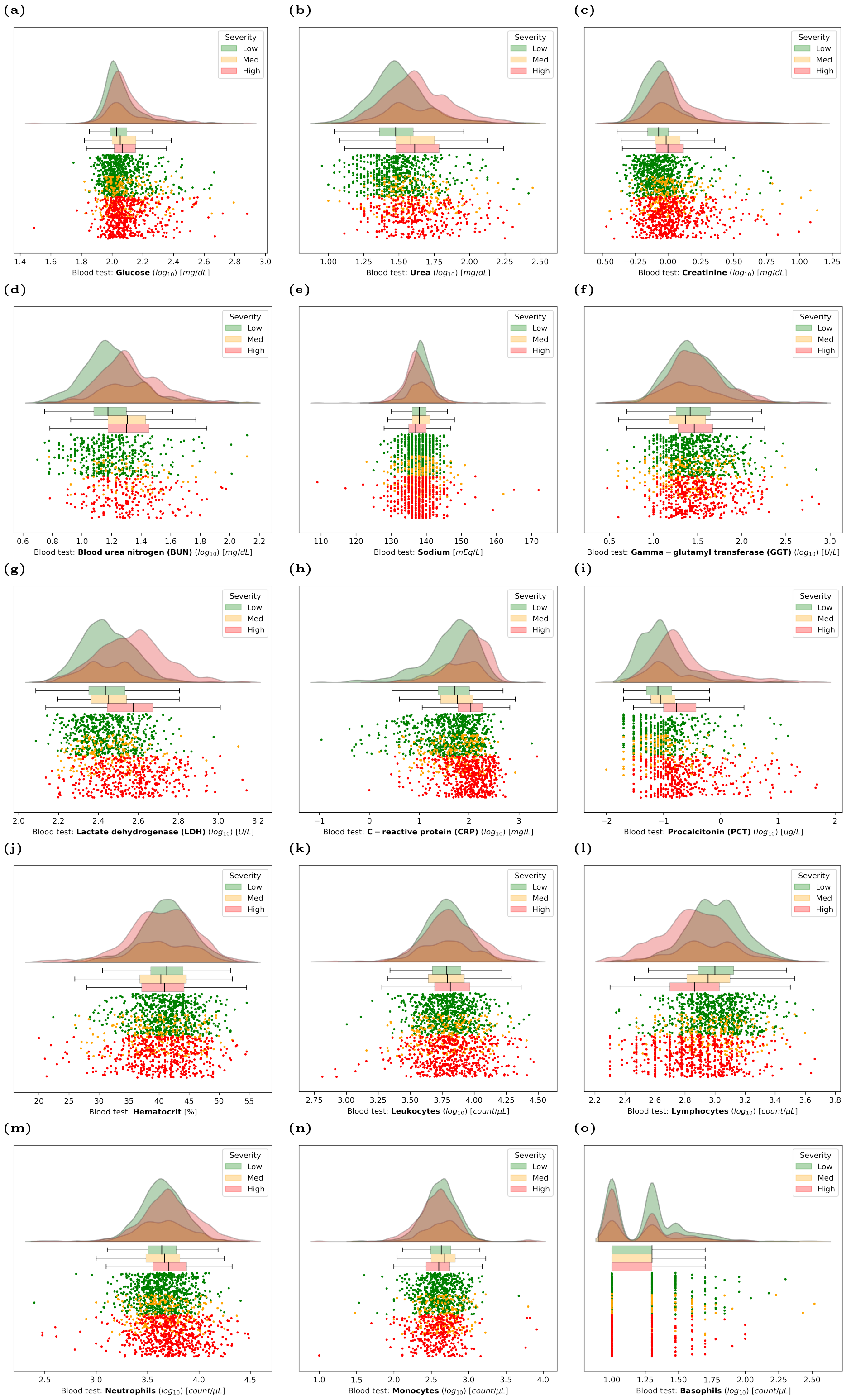

Fig S5. Cohort characteristics (5 of 7). Univariate distributions, by severity.

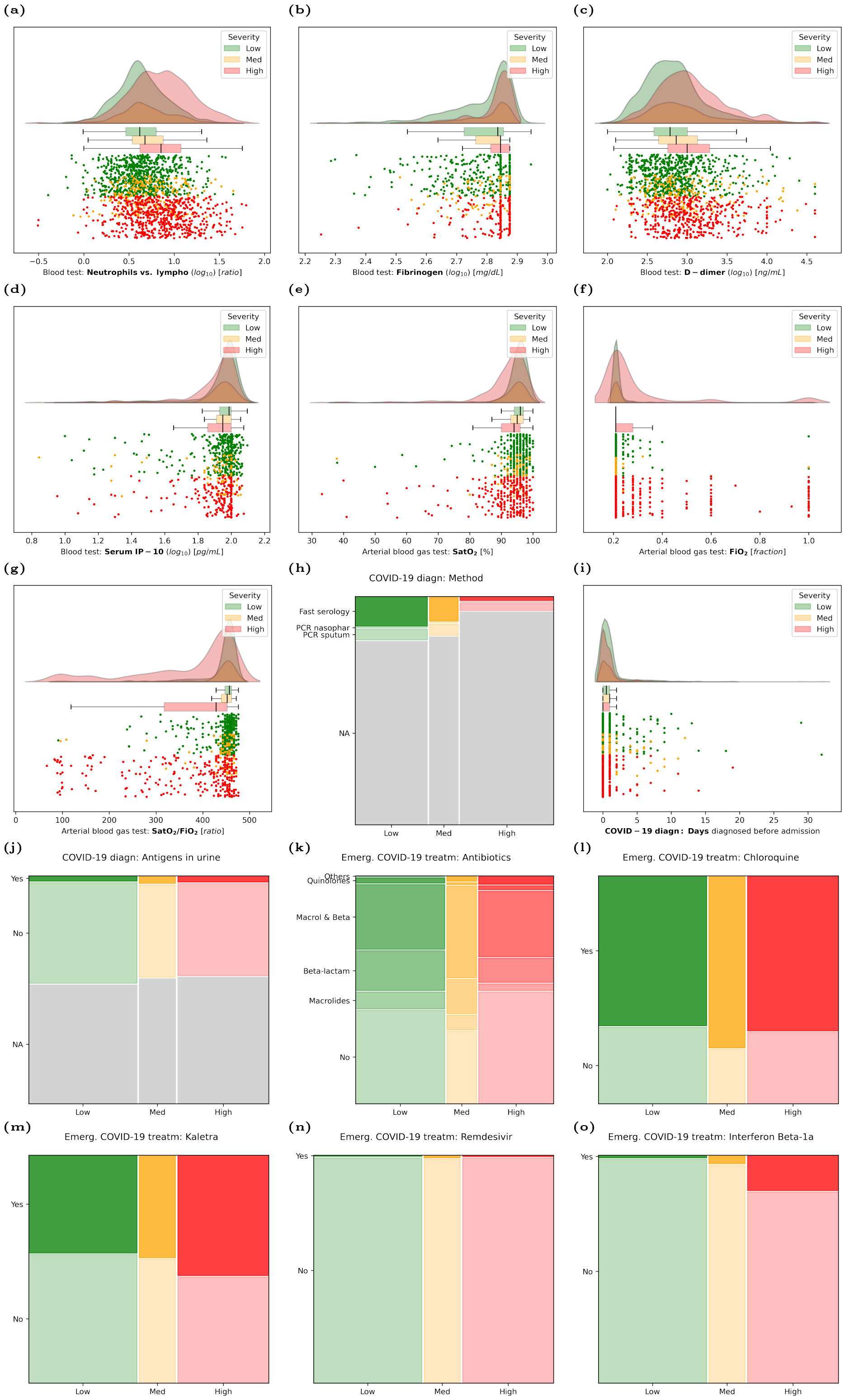

Fig S6. Cohort characteristics (6 of 7). Univariate distributions, by severity.

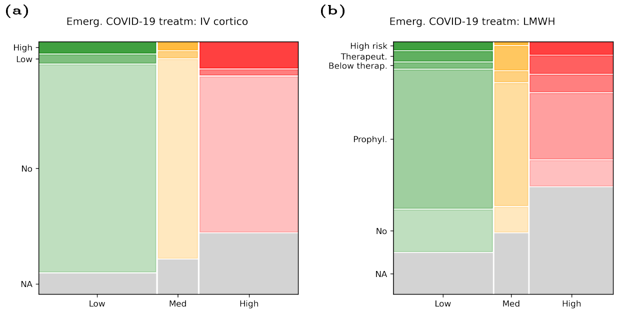

Fig S7. Cohort characteristics (7 of 7). Univariate distributions, by severity.

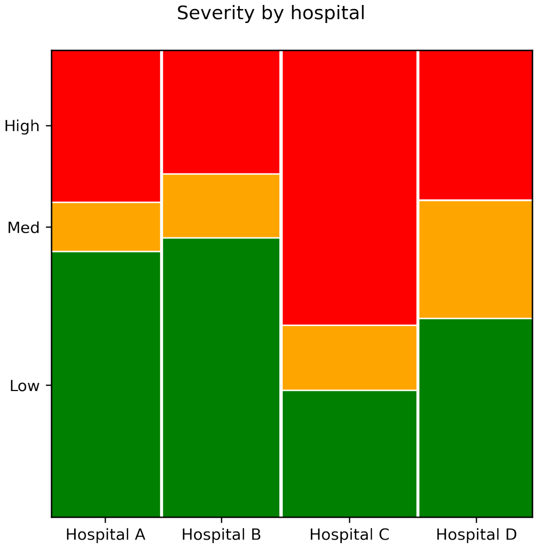

Fig S8. Distribution of cases. By SARS-CoV-2 pneumonia severity, and by hospital.

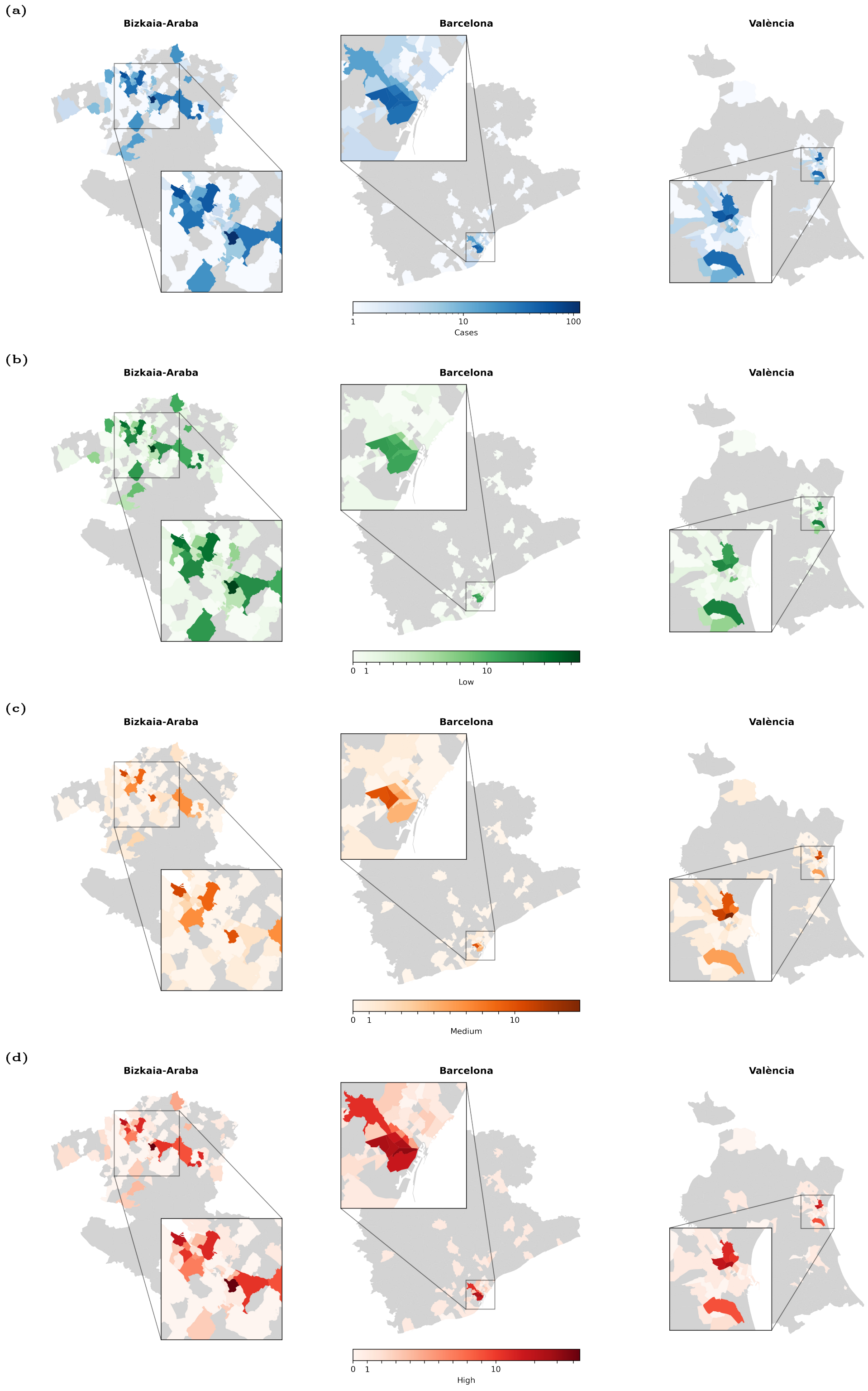

Fig S9. Geographical distributions for the number of cases. *(a)* Total, and by severity group: *(b)* low = green, *(c)* medium = orange, *(d)* high = red. Gray areas represent postcodes without any patient included. Zoomed-in squares focus on the urban zones serviced by the different hospitals.

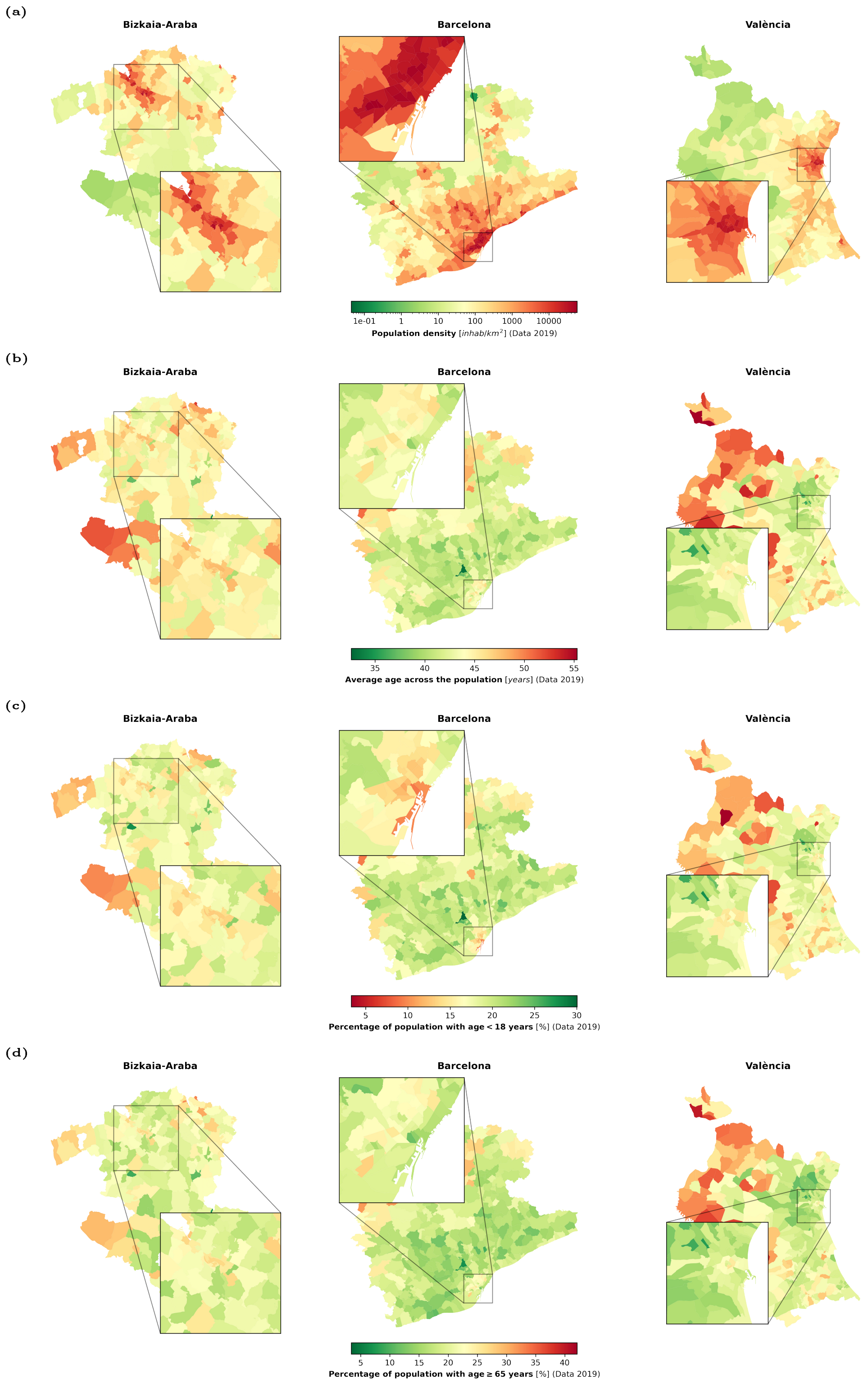

Fig S10. Socioeconomic characteristics per postcode of residence (1 of 2). Sociodemographic and economic situation (2019 census). Zoomed-in squares focus on the urban zones serviced by the different hospitals.

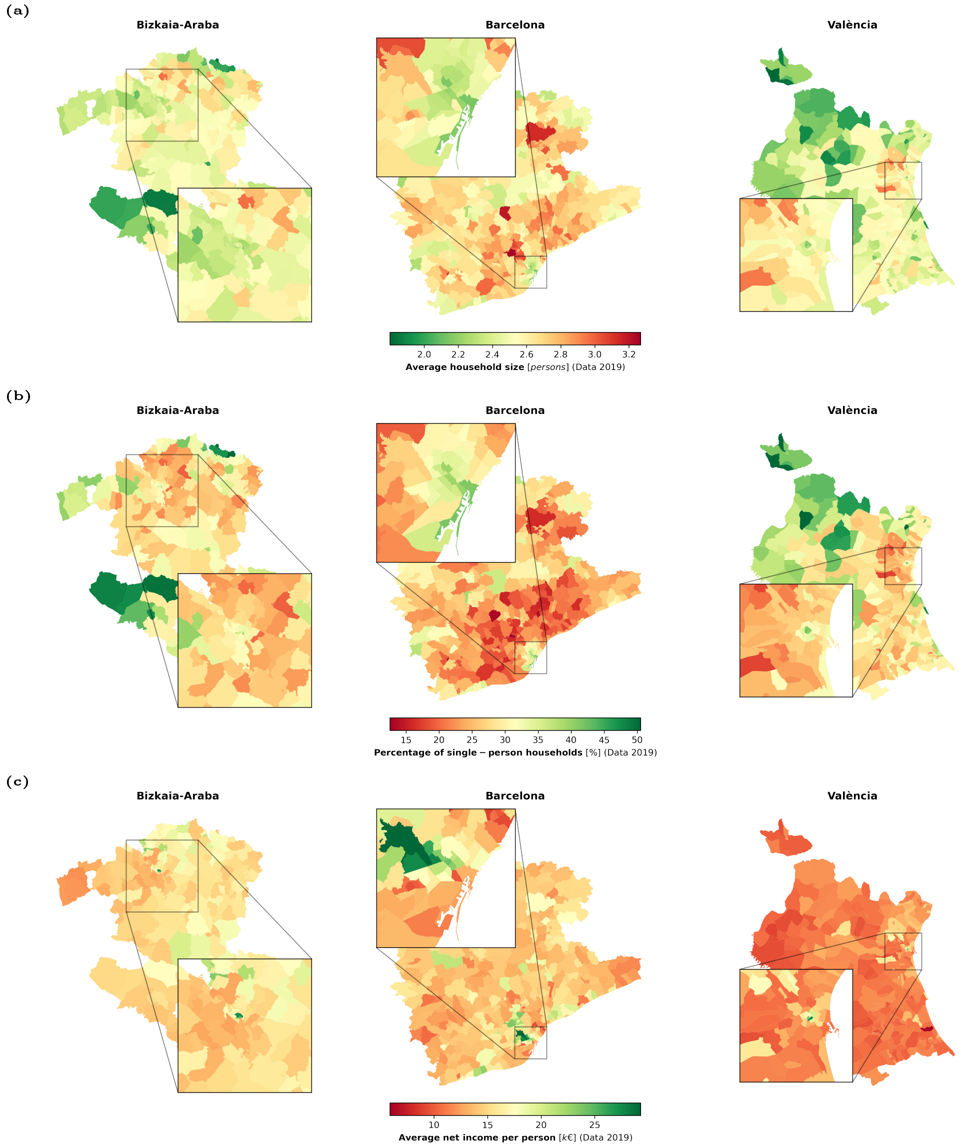

Fig S11. Socioeconomic characteristics per postcode of residence (2 of 2). Sociodemographic and economic situation (2019 census). Zoomed-in squares focus on the urban zones serviced by the different hospitals.

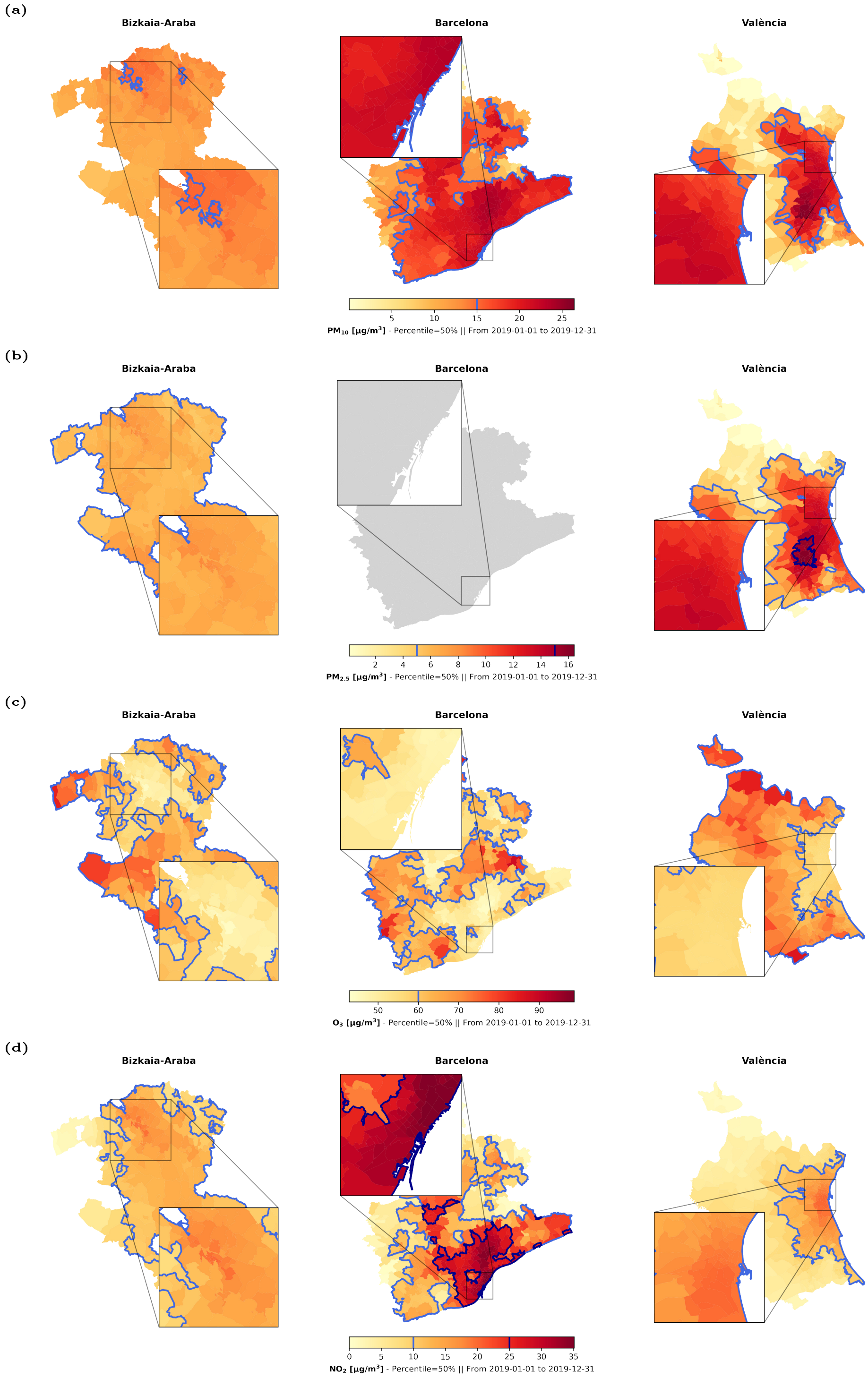

Fig S12. Chronic exposure to air pollution [throughout 2019], per postcode of residence: 50% percentile of daily values (1 of 2); ***(a)*** **PM_{10},** ***(b)*** **$PM_{2.5}$,** ***(c)*** **O_3,** ***(d)*** **NO_2.** Postcodes highlighted with light-blue borders experienced levels above the annual air quality guideline levels (AQG) recommended by the World Health Organization (WHO) [3]; whereas postcodes highlighted in dark-blue exceeded the daily AQG. For *(c)* O_3, AQG levels are respectively peak season and 8-hourly. Catalonia's air quality network did not report *(b)* $PM_{2.5}$ measurements for Barcelona. Zoomed-in squares focus on the urban zones serviced by the different hospitals.

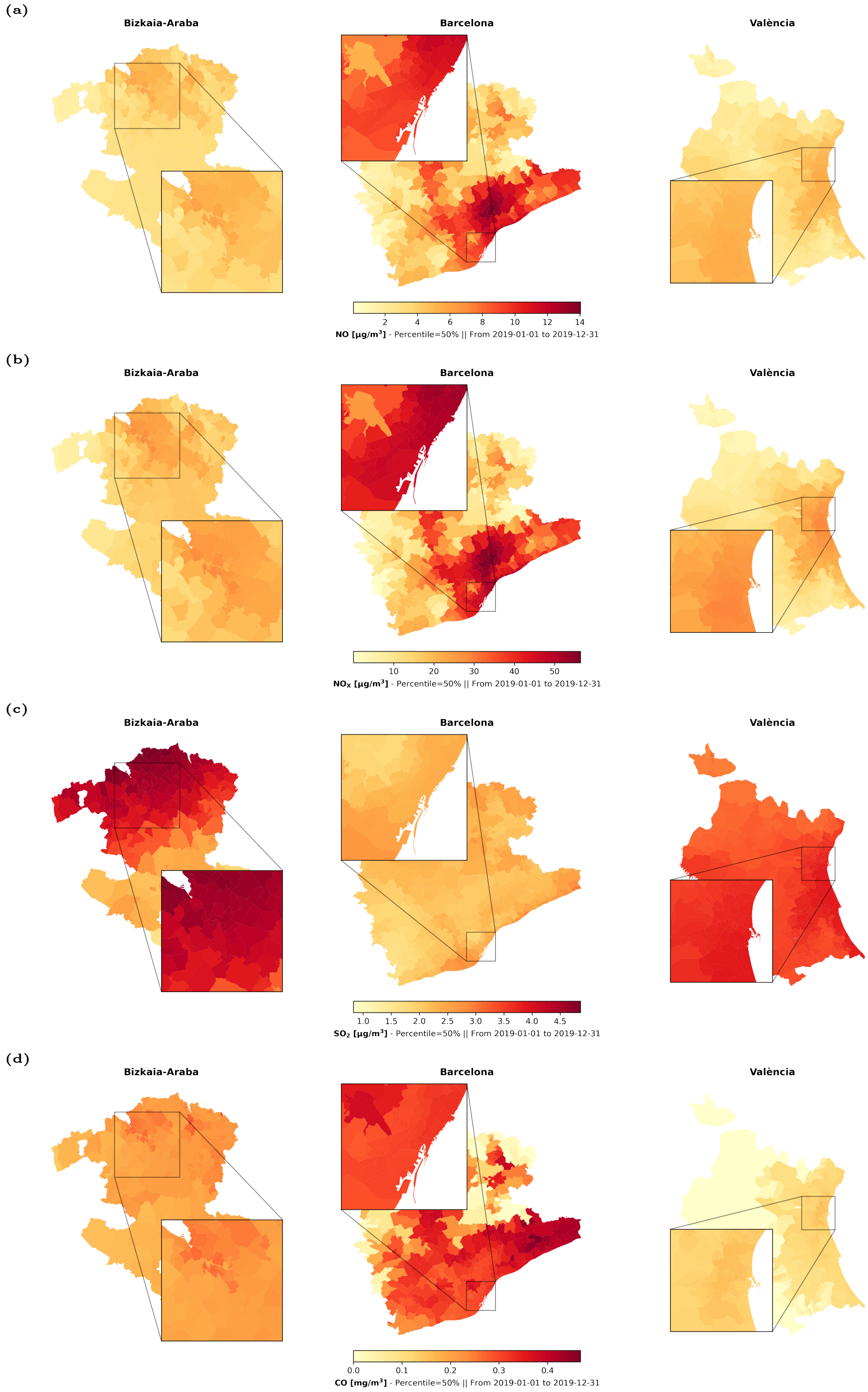

Fig S13. Chronic exposure to air pollution [throughout 2019], per postcode of residence: 50% percentile of daily values (1 of 2); ***(a)*** **NO,** ***(b)*** **NO_X,** ***(c)*** **SO_2,** ***(d)*** **CO.** The WHO does not currently publish recommendations on neither yearly nor daily AQG levels for these pollutants. Zoomed-in squares focus on the urban zones serviced by the different hospitals.

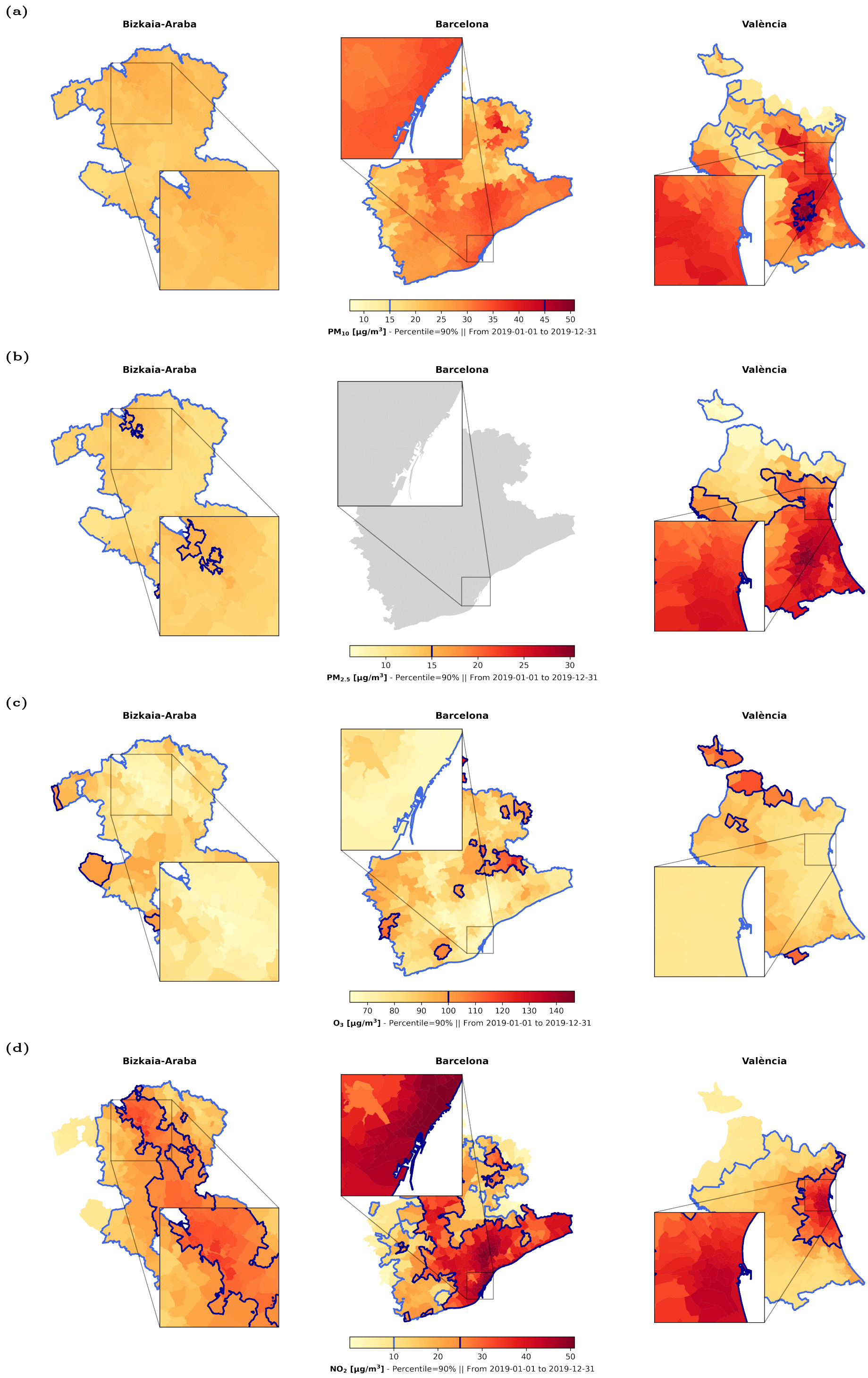

Fig S14. Chronic exposure to air pollution [throughout 2019], per postcode of residence: 90% percentile of daily values (1 of 2); *(a)* PM_{10}, *(b)* $PM_{2.5}$, *(c)* O_3, *(d)* NO_2. Postcodes highlighted with light-blue borders experienced levels above the annual air quality guideline levels (AQG) recommended by the World Health Organization (WHO) [3]; whereas postcodes highlighted in dark-blue exceeded the daily AQG. For *(c)* O_3, AQG levels are respectively peak season and 8-hourly. Catalonia's air quality network did not report *(b)* $PM_{2.5}$ measurements for Barcelona. Zoomed-in squares focus on the urban zones serviced by the different hospitals.

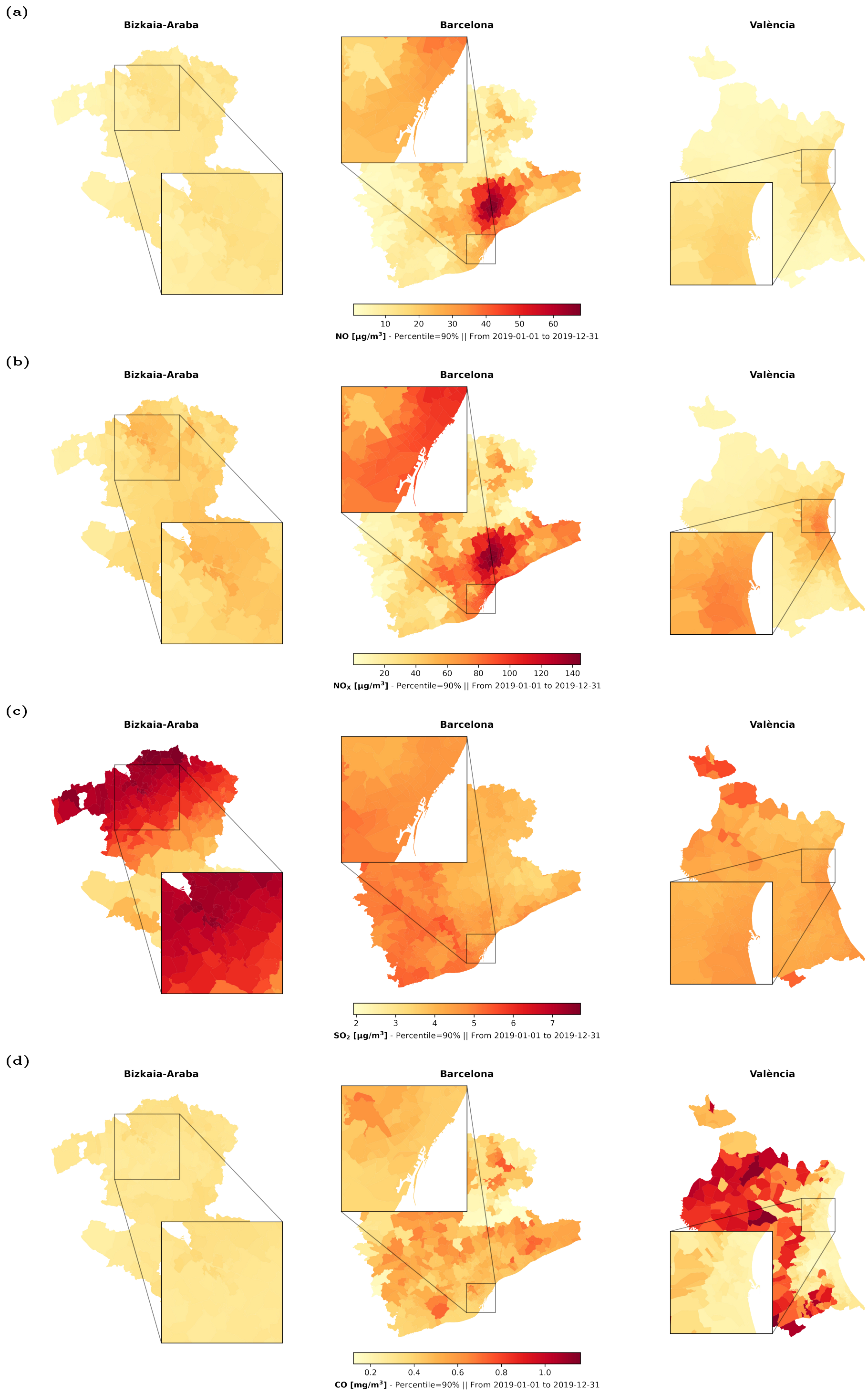

Fig S15. Chronic exposure to air pollution [throughout 2019], per postcode of residence: 90% percentile of daily values (1 of 2); *(a)* **NO,** *(b)* **NO_X,** *(c)* **SO_2,** *(d)* **CO.** The WHO does not currently publish recommendations on neither yearly nor daily AQG levels for these pollutants. Zoomed-in squares focus on the urban zones serviced by the different hospitals.

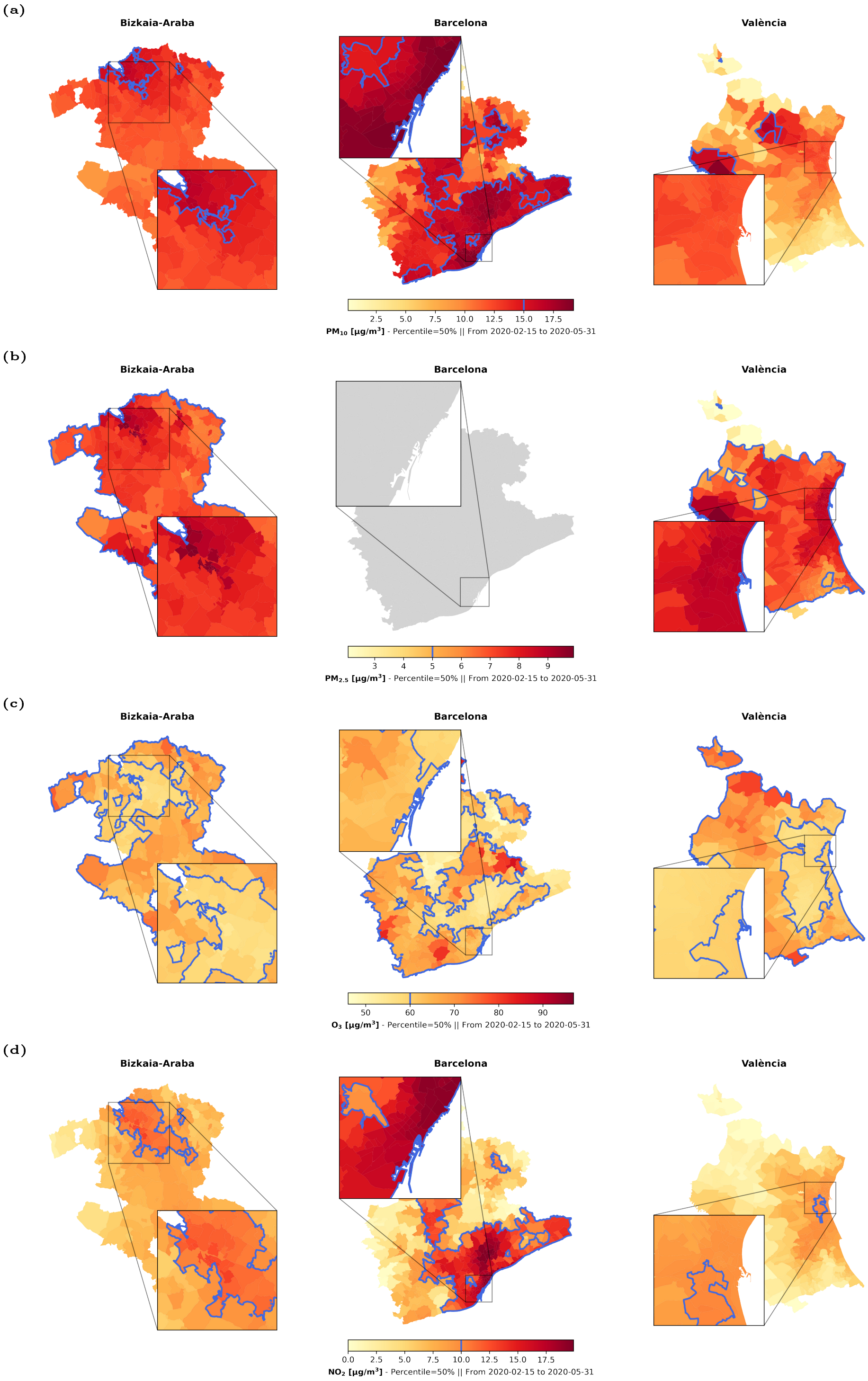

Fig S16. Acute exposure to air pollution [date range from 2020/02/15 to 2020/05/31], per postcode of residence: 50% percentile of daily values (1 of 2); *(a)* PM_{10}, *(b)* $PM_{2.5}$, *(c)* O_3, *(d)* NO_2. Postcodes highlighted with light-blue borders experienced levels above the annual air quality guideline levels (AQG) recommended by the World Health Organization (WHO) [3]; whereas postcodes highlighted in dark-blue exceeded the daily AQG. For *(c)* O_3, AQG levels are respectively peak season and 8-hourly. Catalonia's air quality network did not report *(b)* $PM_{2.5}$ measurements for Barcelona. Zoomed-in squares focus on the urban zones serviced by the different hospitals.

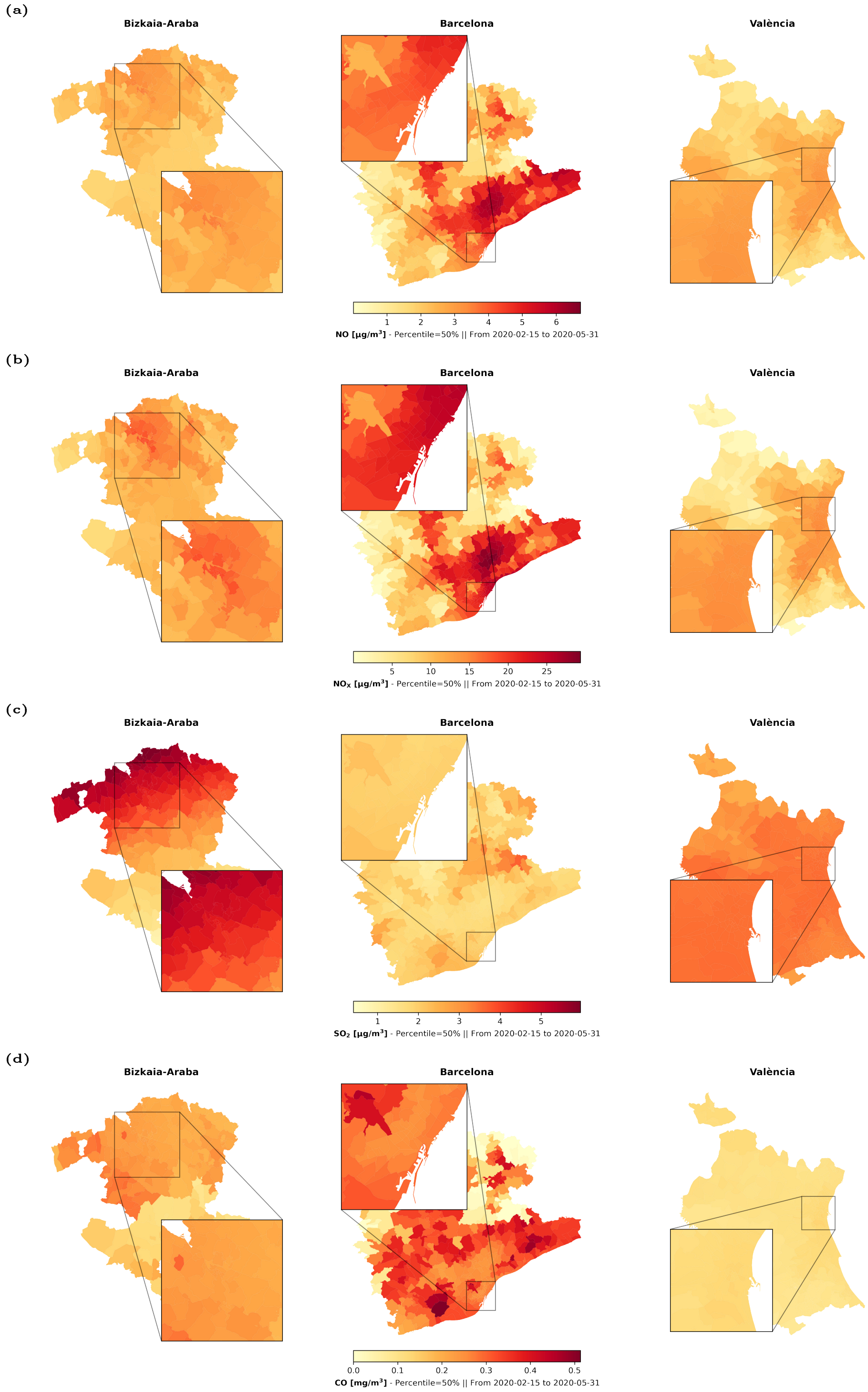

Fig S17. Acute exposure to air pollution [date range from 2020/02/15 to 2020/05/31], per postcode of residence: 50% percentile of daily values (1 of 2); ***(a)*** **NO,** ***(b)*** **NO_X,** ***(c)*** **SO_2,** ***(d)*** **CO.** The WHO does not currently publish recommendations on neither yearly nor daily AQG levels for these pollutants. Zoomed-in squares focus on the urban zones serviced by the different hospitals.

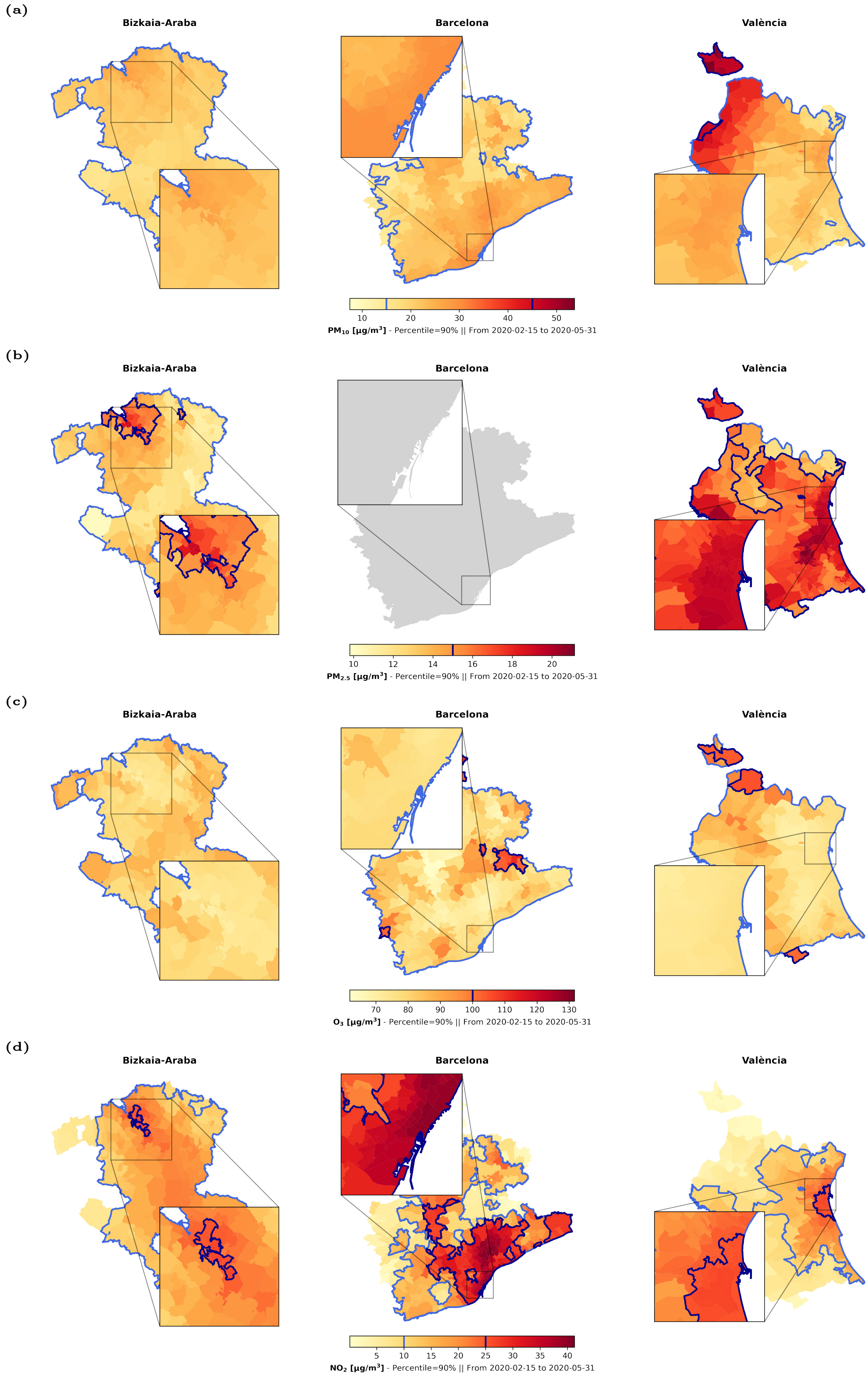

Fig S18. Acute exposure to air pollution [date range from 2020/02/15 to 2020/05/31], per postcode of residence: 90% percentile of daily values (1 of 2); *(a)* PM_{10}, *(b)* $PM_{2.5}$, *(c)* O_3, *(d)* NO_2. Postcodes highlighted with light-blue borders experienced levels above the annual air quality guideline levels (AQG) recommended by the World Health Organization (WHO) [3]; whereas postcodes highlighted in dark-blue exceeded the daily AQG. For *(c)* O_3, AQG levels are respectively peak season and 8-hourly. Catalonia's air quality network did not report *(b)* $PM_{2.5}$ measurements for Barcelona. Zoomed-in squares focus on the urban zones serviced by the different hospitals.

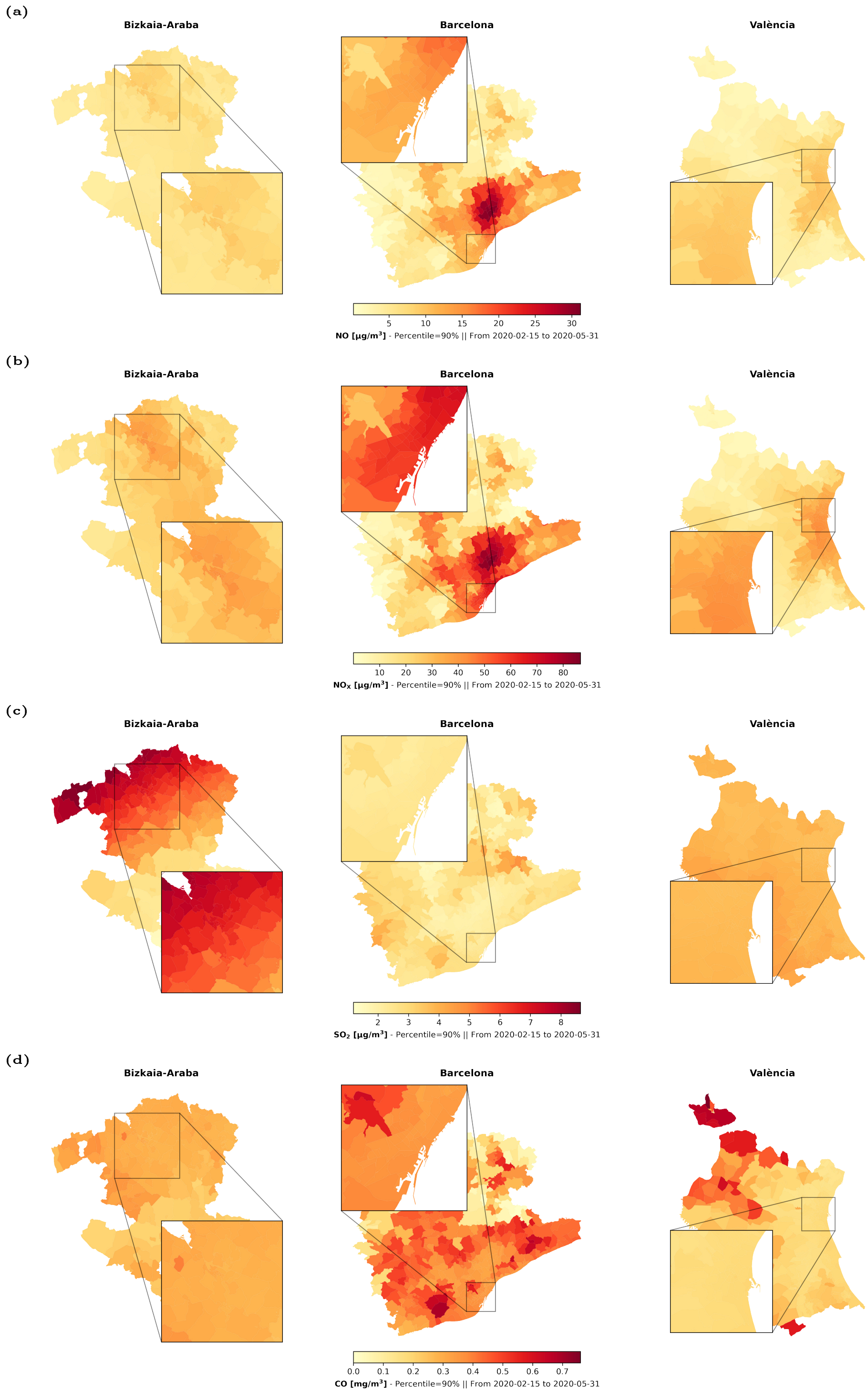

Fig S19. Acute exposure to air pollution [date range from 2020/02/15 to 2020/05/31], per postcode of residence: 90% percentile of daily values (1 of 2); ***(a)*** **NO,** ***(b)*** **NO_X,** ***(c)*** **SO_2,** ***(d)*** **CO.** The WHO does not currently publish recommendations on neither yearly nor daily AQG levels for these pollutants. Zoomed-in squares focus on the urban zones serviced by the different hospitals.

S.C Methods: Hyperparameters

S.C.1 Hyperparameters for the ‘wrapped’ estimators

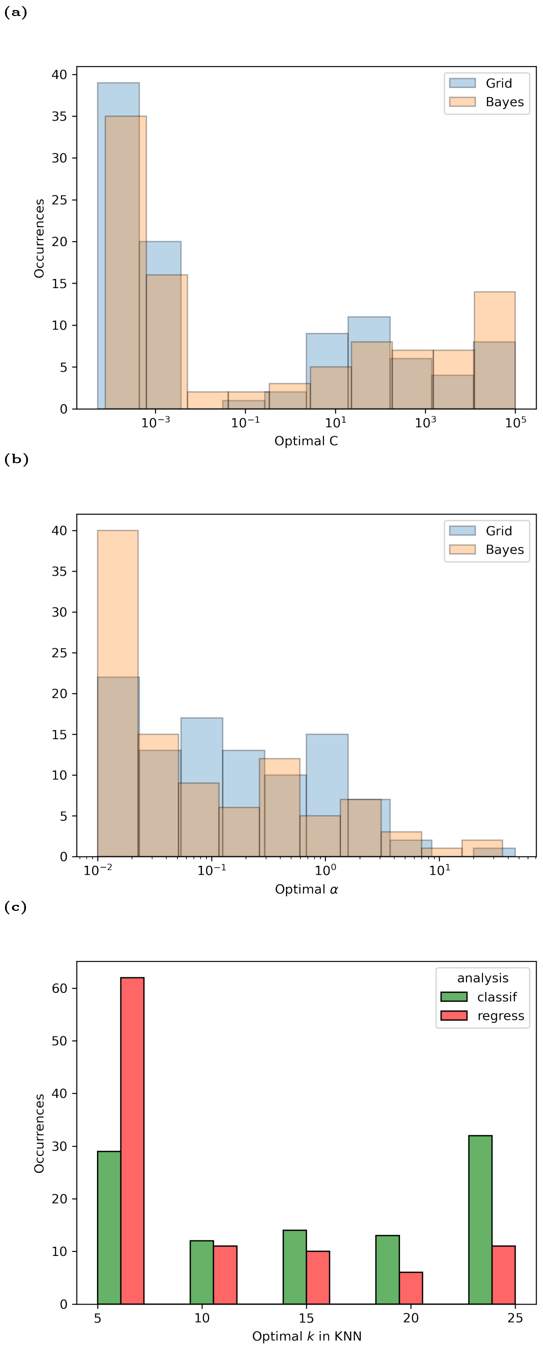

Fig S20. Histogram of optimal hyperparameter choices for linear and nearest neighbor estimators, across M=100 bootstrap samples. *(a)* Optimal regularization parameter C for L^2-norm penalized logistic regression (LR), tuned by grid and Bayesian search strategies. *(b)* Optimal regularization parameter α for L^2-penalized Ridge regression, also tuned by grid and Bayesian search. *(b)* Optimal number of neighbors k, for k-nearest neighbor classification and regression –ordinal regression approach [4]–, tuned by grid search.

S.C.2 Hyperparameters for GA and BPSO search

For the search based on Genetic Algorithms (GA), we fixed: a ‘canonical’ population size with n_{pop}=100 individuals, an intermediate crossover rate of p_{cx}=0.70, and two mutation probabilities: p_m=0.001 (for a faster convergence), and p_m=0.020 (for a more exploratory algorithm). In our analyses, we opted for a $k$nn imputer and linear models (L^2-LR, Ridge), with M=10 bootstrap samples, and studied how many generations n_{gen} were required to reach convergence (Fig S21).

In addition, for the search based on Binary Particle Swarm Optimization (BPSO), we conducted similar analyses with the same wrapped models. Having fixed certain BPSO’s parameters to ‘canonical’ values, namely: ϕ_p, ϕ_g=0.5, n_{pop}=30, and n_{gen}=2000; we explored the combinations between values $|v|_{\max}$=2 or $|v|_{\max}$=6, and ω=0.6 or ω=0.9 (Fig S22).

For further details, the interested reader is referred to [5] [Section 5.3.2].

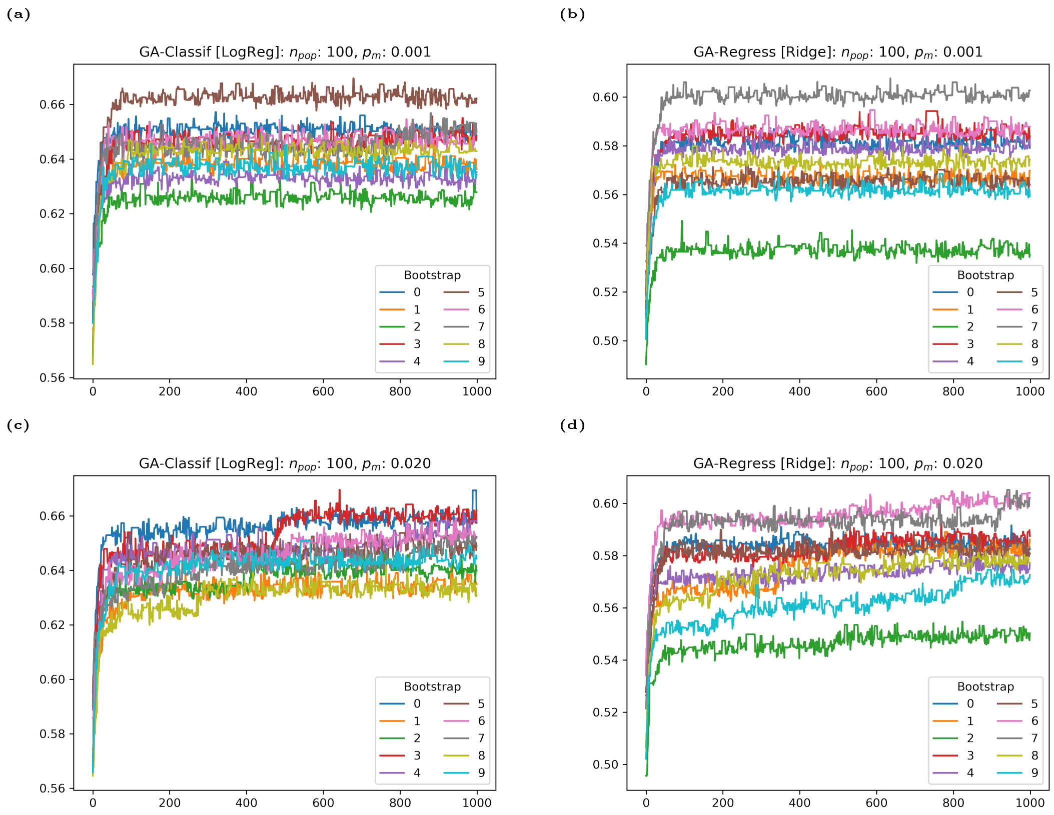

Fig S21. GA convergence – Fitness curves along iterations, for M=10 bootstrap samples. On the left *(a, c)*: wrapped L^2-LR classifier; on the right *(b, d)*: wrapped Ridge regressor. Top row *(a, b)*: genetic mutation probability p_m=0.001; bottom row *(c, d)*: genetic mutation probability p_m=0.020.

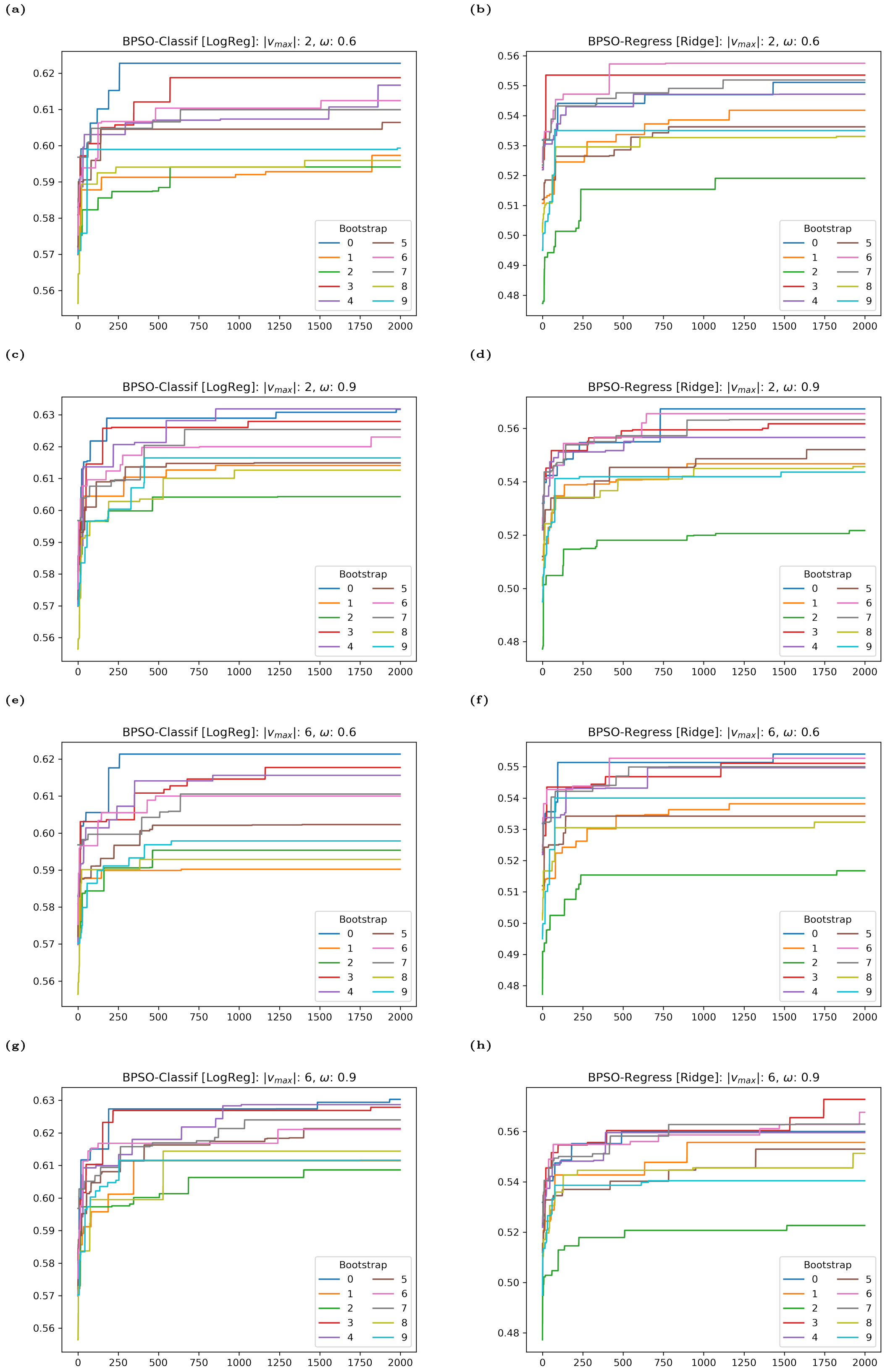

Fig S22. BPSO convergence – Fitness curves along iterations, for M=10 bootstrap samples. On the left *(a, c, e, g)*: wrapped L^2-LR classifier; on the right *(b, d, f, h)*: wrapped Ridge regressor. Top two rows *(a–d)*: $|v|_{\max}$=2; bottom two rows *(e–h)*: $|v|_{\max}$=6. Odd rows *(a, b, e, f)*: ω=0.6; even rows *(c, d, g, h)*: ω=0.9.

S.D Results: Stability

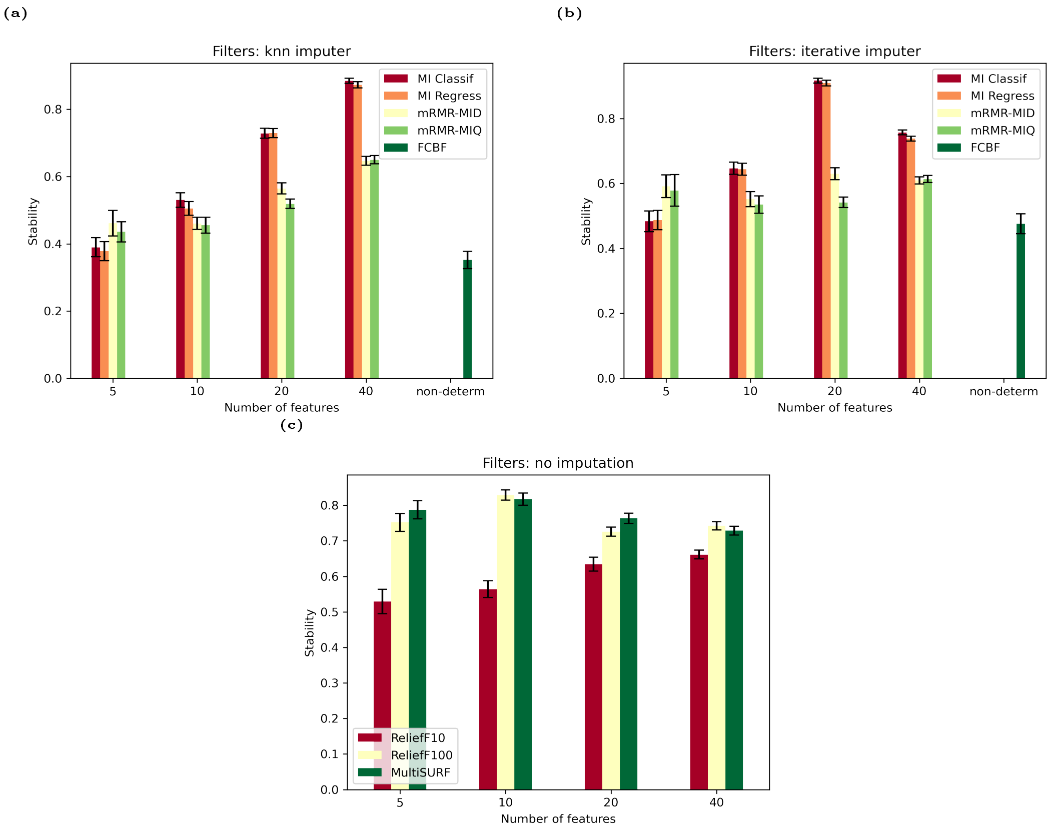

Fig S23. Stability for the filter algorithms. Mean and 95% CI: *(a)* Mutual information (MI)-based, minimum redundancy–maximum relevance (mRMR) and fast correlation-based filter (FCBF), with $k$nn imputation; *(b)* MI, mRMR and FCBF, with iterative imputation; and *(c)* ReliefF and MultiSURF filters, without imputation.

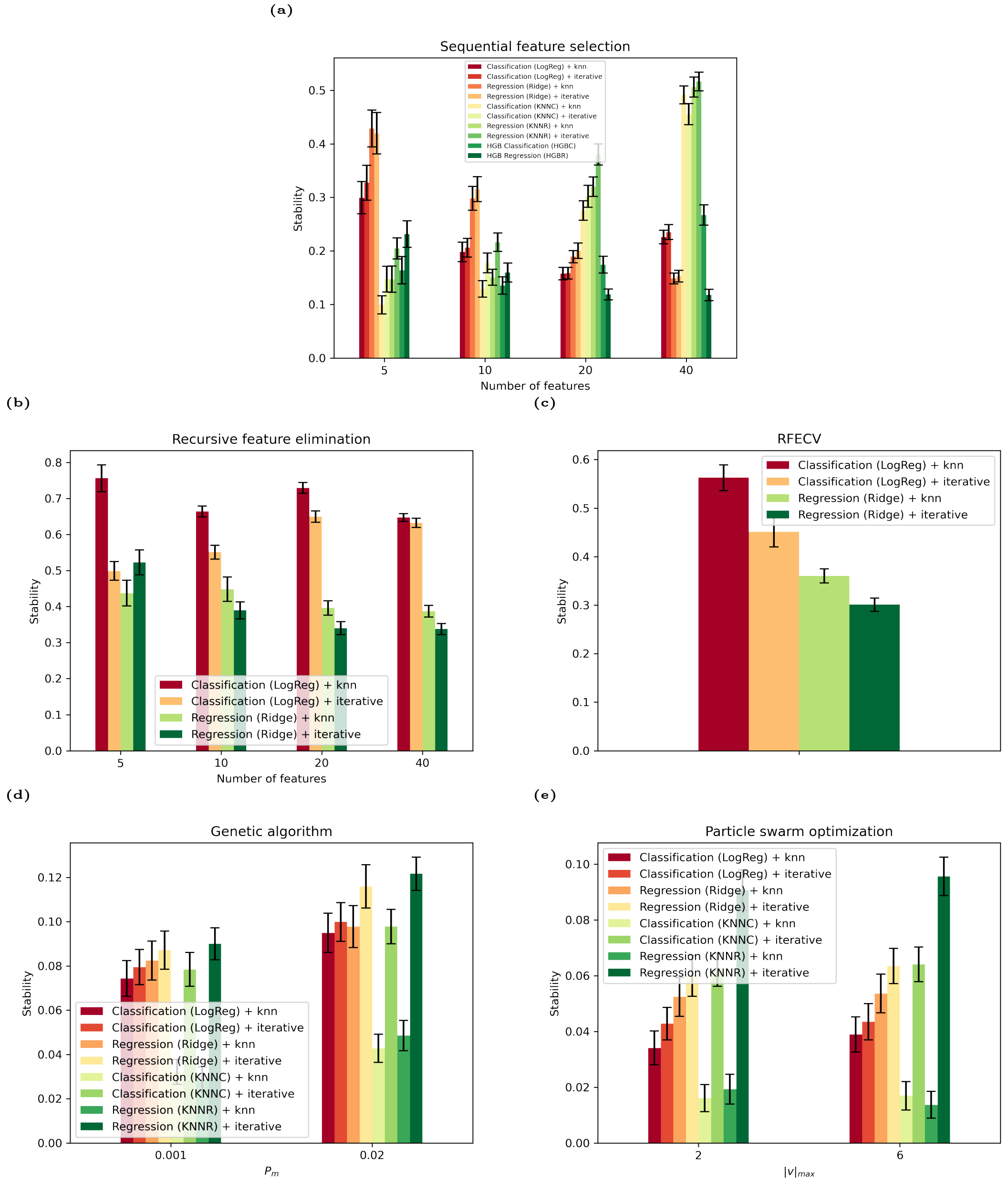

Fig S24. Stability for the wrapper algorithms. Mean and 95% CI.

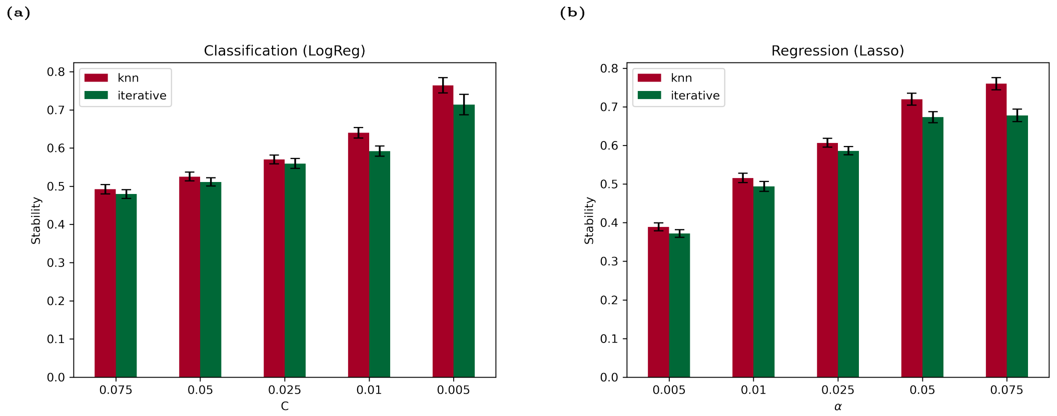

Fig S25. Stability for the embedded algorithms. Mean and 95% CI.

S.E Results: Computation times

The following tables S3, S4 and S5 summarize the computation times required for each FS scenario. Please note that there might be minor inconsistencies, due to an upgrade in our computer cluster's task management software.

Table S3. Computation time for the filter algorithms.
Mean and 95% confidence interval (CI) *[seconds]*.

Imputer	FS: Filter		Number of features to select				
			n_{FS}=5	n_{FS}=10	n_{FS}=20	n_{FS}=40	Not pre-fixed
$k$nn	MI	Classif.	4.89 [4.84, 4.94]	6.1404 [6.08, 6.20]	23.82 [22.16, 25.48]	26.79 [24.82, 28.76]	—
		Regress.	4.84 [4.79, 4.89]	5.8992 [5.84, 5.96]	92.04 [86.27, 97.81]	120.19 [112.94, 127.44]	—
	mRMR	MID	4.86 [4.82, 4.91]	5.91 [5.86, 5.96]	345.62 [325.40, 365.84]	486.24 [456.72, 515.76]	—
		MIQ	4.85 [4.81, 4.90]	5.89 [5.83, 5.95]	1365.40 [1278.22, 1452.57]	1232.83 [1158.39, 1307.28]	—
	FCBF	δ=0	—	—	—	—	2.26 [2.22, 2.30]
Iterat.	MI	Classif.	4.88 [4.83, 4.93]	6.20 [6.12, 6.28]	11.69 [10.55, 12.82]	14.97 [13.44, 16.50]	—
		Regress.	4.86 [4.81, 4.91]	5.98 [5.90, 6.06]	43.70 [37.94, 49.47]	52.67 [44.80, 60.53]	—
	mRMR	MID	4.86 [4.81, 4.92]	6.01 [5.94, 6.08]	281.89 [260.64, 303.14]	417.0 [389.24, 444.82]	—
		MIQ	4.85 [4.80, 4.91]	7.00 [6.77, 7.23]	1430.93] [1346.54, 1515.31]	1185.66 [1114.94, 1256.39]	—
	FCBF	δ=0	—	—	—	—	2.58 [2.51, 2.64]
None	ReliefF	k=10	311.93 [308.71, 315.16]	308.03 [305.13, 310.94]	314.47 [312.09, 316.84]	317.51 [314.51, 320.51]	—
		k=100	548.56 [539.01, 558.11]	582.35 [571.80, 592.89]	585.01 [574.69, 595.33]	569.64 [558.93, 580.35]	—
	MultiSURF		987.60 [969.00, 1006.21]	1009.30 [990.15, 1028.45]	1003.16 [984.74, 1021.57]	1003.56 [985.35, 1021.78]	—

Table S4. Computation time for the wrapper algorithms.
Mean and 95% confidence interval (CI) *[seconds]*.

Imputer	FS: Wrapper			Number of features to select				
	Search	Params	ML	n_{FS}=5	n_{FS}=10	n_{FS}=20	n_{FS}=40	Not pre-fixed
$k$nn	SFS	Forward	L^2-LR	158.11 [155.42, 160.81]	325.11 [319.60, 330.62]	679.03 [666.67, 691.40]	1465.19 [1435.45, 1494.94]	—
			Ridge	18.96 [18.42 ,19.51]	37.95 [37.12, 38.77]	76.54 [74.95, 78.12]	147.86 [144.86, 150.86]	—
			kNNC	54.62 [53.49, 55.75]	165.93 [162.59, 169.27]	408.67 [400.20, 417.15]	671.74 [657.15, 686.34]	—
			kNNR	51.96 [50.93, 52.99]	160.31 [157.13, 163.49]	396.36 [387.70, 405.01]	653.26 [639.93, 666.59]	—
	RFE	—	L^2-LR	51.09 [49.68, 52.50]	50.77 [49.33, 52.22]	49.86 [48.39, 51.32]	46.90 [45.41, 48.39]	—
			Ridge	1.26 [1.25, 1.27]	1.25 [1.23, 1.26]	1.19 [1.18, 1.21]	1.08 [1.06, 1.09]	—
	RFECV	—	L^2-LR	—	—	—	—	55.87 [54.25, 57.49]
			Ridge	—	—	—	—	1.58 [1.56, 1.61]
	GA	p_m=0.001	L^2-LR	—	—	—	—	11335.24 [10984.42, 11686.05]
			Ridge	—	—	—	—	1003.79[984.11,1023.47]
			kNNC	—	—	—	—	4882.58 [4589.46, 5175.71]
			kNNR	—	—	—	—	5316.95 [5006.60, 5627.31]
		p_m=0.020	L^2-LR	—	—	—	—	44841.32 [38110.01, 51572.62]
			Ridge	—	—	—	—	1932.29[1893.14,1971.44]
			kNNC	—	—	—	—	9600.13 [9087.31, 10112.95]
			kNNR	—	—	—	—	12510.16 [11333.43, 13686.88]
	BPSO	ω=2	L^2-LR	—	—	—	—	57560.82 [53521.07, 61600.57]
			Ridge	—	—	—	—	1899.10 [1850.75, 1947.45]
			kNNC	—	—	—	—	7062.07 [6884.91, 7239.24]
			kNNR	—	—	—	—	7882.23 [7301.65, 8462.81]
		ω=6	L^2-LR	—	—	—	—	30664.24 [30025.31, 31303.17]
			Ridge	—	—	—	—	1894.33 [1846.82, 1941.83]
			kNNC	—	—	—	—	20686.65 [15260.81, 26112.48]
			kNNR	—	—	—	—	6676.90 [6499.71, 6854.10]
Iterat.	SFS	Forward	L^2-LR	158.42 [155.17, 161.68]	324.07 [317.69, 330.44]	680.59 [666.00, 695.19]	1465.61 [1435.25, 1495.98]	—
			Ridge	18.69 [18.13, 19.25]	38.28 [37.33, 39.22]	74.99 [73.26, 76.72]	149.08 [145.82, 152.34]	—
			kNNC	54.91 [53.68, 56.13]	169.28 [165.45, 173.12]	423.21 [413.59, 432.83]	721.44 [704.99, 737.88]	—
			kNNR	52.21 [51.17, 53.24]	162.95 [159.22, 166.69]	406.14 [397.31, 414.96]	707.01 [691.11, 722.91]	—
	RFE	—	L^2-LR	47.06 [45.79, 48.34]	47.10 [45.71, 48.49]	45.00 [43.73, 46.27]	42.97 [41.67, 44.28]	—
			Ridge	1.27 [1.25, 1.29]	1.25 [1.23, 1.27]	1.22 [1.19, 1.24]	1.08 [1.07, 1.10]	—
	RFECV	—	L^2-LR	—	—	—	—	52.65 [51.09, 54.20]
			Ridge	—	—	—	—	1.72 [1.68, 1.76]
	GA	p_m=0.001	L^2-LR	—	—	—	—	11420.93 [11136.89, 11704.97]
			Ridge	—	—	—	—	1004.72 [984.82, 1024.62]
			kNNC	—	—	—	—	5438.08 [5153.70, 5722.46]
			kNNR	—	—	—	—	5980.13 [5666.61, 6293.65]
		p_m=0.020	L^2-LR	—	—	—	—	22198.53 [21667.22, 22729.85]
			Ridge	—	—	—	—	1934.62 [1895.81, 1973.42]
			kNNC	—	—	—	—	15950.16 [12732.78, 19167.54]
			kNNR	—	—	—	—	13157.30 [12293.62, 14020.99]
	BPSO	ω=2	L^2-LR	—	—	—	—	27190.00 [26628.77, 27751.23]
			Ridge	—	—	—	—	1909.92 [1860.91, 1958.92]
			kNNC	—	—	—	—	7990.36 [7790.55, 8190.17]
			kNNR	—	—	—	—	8228.20 [7921.83, 8534.56]
		ω=6	L^2-LR	—	—	—	—	27208.27 [26582.05, 27834.50]
			Ridge	—	—	—	—	1907.52 [1858.67, 1956.37]
			kNNC	—	—	—	—	8305.87 [8156.78, 8454.96]
			kNNR	—	—	—	—	7516.47 [7326.43, 7706.51]
None	SFS	Forward	HGBC	2111.75 [2046.20, 2177.29]	5389.47 [5286.61, 5492.33]	11103.46 [10888.53, 11318.38]	20610.98 [20232.74, 20989.22]	—
			HGBR	788.59 [774.47, 802.71]	1945.42 [1911.20, 1979.64]	4310.54 [4236.38, 4384.71]	9110.04 [8956.50, 9263.58]	—
	GA	p_m	HGBC	—	—	—	—	Exceeds max. runtime*
			HGBR	—	—	—	—	Exceeds max. runtime*
	BPSO	ω	HGBC	—	—	—	—	Exceeds max. runtime*
			HGBR	—	—	—	—	Exceeds max. runtime*

*Computations for GA and BPSO with ‘wrapped’ HGB estimators were discarded, as they exceeded the maximum runtime of 1 day (86,400 s) per bootstrap sample.

Table S5. Computation time for the embedded algorithms.
Mean and 95% confidence interval (CI) *[seconds]*.

**Imputer**	**FS: Embedded**		**Num. feats:** Not pre-fixed
$k$nn	L^1-LR	C=0.075	8.09 [7.58, 8.59]
		C=0.050	5.81 [5.42, 6.21]
		C=0.025	3.61 [3.28, 3.93]
		C=0.010	1.20 [1.14, 1.26]
		C=0.005	0.58 [0.55, 0.60]
	Lasso	α=0.005	0.04 [0.04, 0.04]
		α=0.010	0.03 [0.03, 0.03]
		α=0.025	0.02 [0.02, 0.02]
		α=0.050	0.02 [0.02, 0.02]
		α=0.075	0.02 [0.02, 0.02]
Iterat.	L^1-LR	C=0.075	4.94 [4.63, 5.26]
		C=0.050	3.45 [3.25, 3.65]
		C=0.025	2.70 [2.30, 3.10]
		C=0.010	0.69 [0.62, 0.75]
		C=0.005	0.29 [0.28, 0.30]
	Lasso	α=0.005	0.03 [0.03, 0.03]
		α=0.010	0.02 [0.02, 0.03]
		α=0.025	0.02 [0.02, 0.02]
		α=0.050	0.01 [0.01, 0.01]
		α=0.075	0.01 [0.01, 0.01]

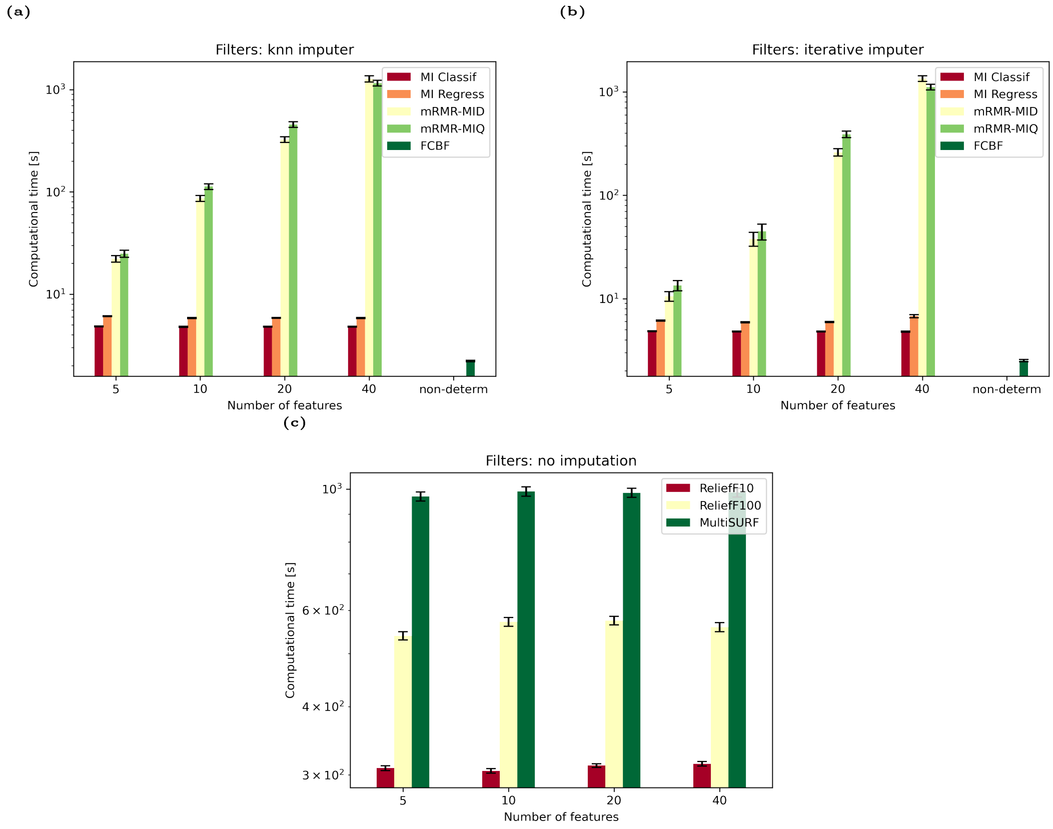

Fig S26. Computation time for the filter algorithms. Mean and 95% CI *[seconds]*: *(a)* Mutual information (MI)-based, minimum redundancy–maximum relevance (mRMR) and fast correlation-based filter (FCBF), with $k$nn imputation; *(b)* MI, mRMR and FCBF, with iterative imputation; and *(c)* ReliefF and MultiSURF filters, without imputation.

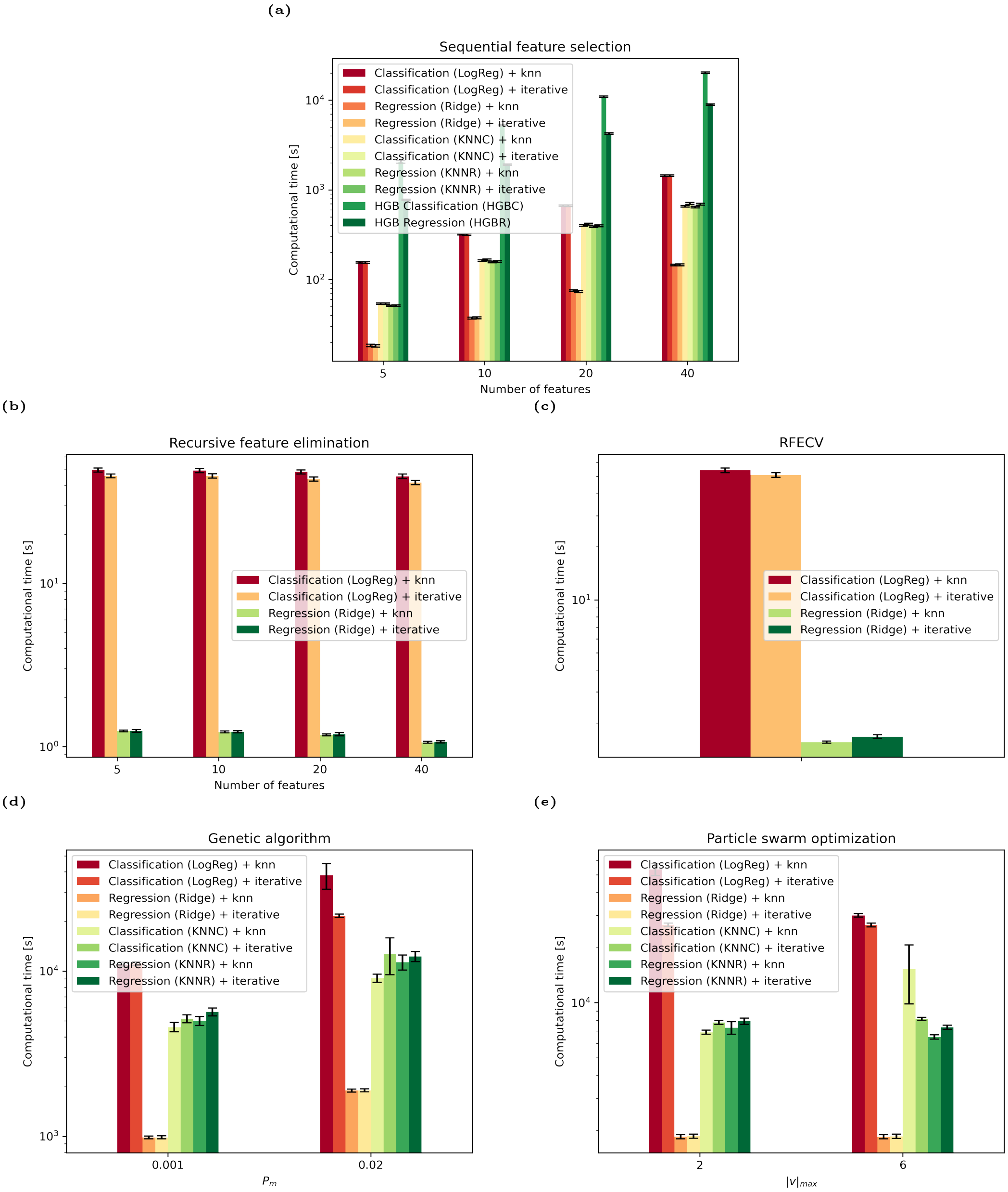

Fig S27. Computation time for the wrapper algorithms. Mean and 95% CI *[seconds]*.

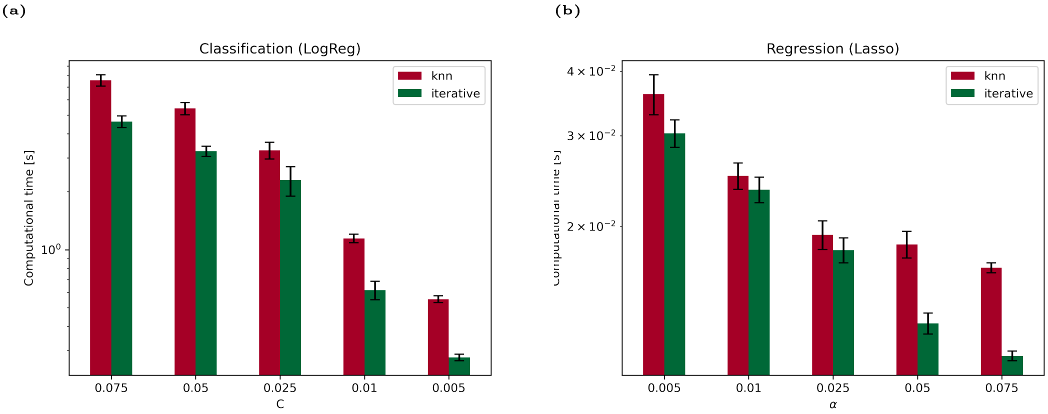

Fig S28. Computation time for the embedded algorithms. Mean and 95% CI *[seconds]*.

References

[1] Bergsma W. A bias-correction for Cramér's V and Tschuprow's T. Journal of the Korean Statistical Society. 2013;42(3):323–328. doi:10.1016/j.jkss.2012.10.002.

[2] Cohen J. Statistical power analysis for the behavioral sciences. Routledge; 2013.

[3] World Health Organization. WHO global air quality guidelines: particulate matter ($PM_{2.5}$ and PM_{10}), ozone, nitrogen dioxide, sulfur dioxide and carbon monoxide. World Health Organization; 2021. Available from: `https://apps.who.int/iris/handle/10665/345329`.

[4] Gutiérrez PA, Pérez-Ortiz M, Sánchez-Monedero J, Fernández-Navarro F, Hervás-Martínez C. Ordinal regression methods: Survey and experimental study. IEEE Trans Knowl Data Eng. 2016;28(1):127–146. doi:10.1109/TKDE.2015.2457911.

[5] Hayet-Otero M. Variable selection in high-dimensional data: application in a SARS-CoV-2 pneumonia clinical data-set [MSc Thesis]. University of the Basque Country (UPV/EHU); 2021. Available from: `https://bird.bcamath.org/handle/20.500.11824/1537`.
