# Supplementary material for: Extracting relevant predictive variables for COVID-19 severity prognosis: An exhaustive comparison of feature selection techniques
Source: PLoS One. 2023 Apr 13;18(4):e0284150. doi: 10.1371/journal.pone.0284150 (PMC10101453; doi:10.1371/journal.pone.0284150)
Supplement: Attachment — Submitted filename: Response to Reviewers.pdf [file pone.0284150.s002.pdf]

Editorial Board – PLOS ONE
December 20th, 2022

Dear PLOS ONE Academic Editor, **Dr. Sathishkumar V E**:

First, we would like to take this opportunity to thank you, as well as both reviewers, for the effort in assessing our article *“Extracting relevant predictive variables for COVID-19 severity prognosis: An exhaustive comparison of feature selection techniques”*; as well as for the valuable, detailed and insightful comments provided. We have taken your feedback in careful consideration and we have worked our best to address the issues raised by them, and thus to enhance the quality of our manuscript.

We are glad to resubmit our revised draft, hoping that our edits and responses below have addressed satisfactorily all the concerns.

To facilitate your review of our revisions, **the following pages contain a point-by-point response to the questions and remarks** delivered in your decision letter dated November 7th, 2022.

The revised manuscript reflects our edits with respect to the original version, highlighted in a colour-coded manner, to identify and track the source of the remark. In **blue**, we address **requests by Reviewer #1**; in **green**, by **Reviewer #2**; in **magenta**, changes to adhere to **PLOS ONE’s formatting requirements**; and finally in **cyan, some clarifications** which we felt contribute to an improved readability of our work.

On behalf of all my co-authors, I thank you again for your feedback and for giving us the chance to revise our manuscript, and (we are positive) to have improved its scientific quality.

We look forward to hearing from you at your convenience.

Sincerely,

Dr. Fernando García-García
Postdoc fellow, Applied Statistics
Basque Center for Applied Mathematics (BCAM)
Alameda de Mazarredo, 14
48009 – Bilbao, Basque Country, Spain
+34 946 567 842
fegarcia@bcamath.org

(Continues in the next page)

PLOS ONE journal requirements:

1. Please ensure that your manuscript meets PLOS ONE's style requirements, including those for file naming.

In order to comply with PLOS ONE's style requirements, we have made the following changes:

- Updated the names of all files (including manuscript and figures).
- Removed the footnote in page 5, as they are not allowed.
- Adapted the table and figure captions, to include a caption in the required format.

2. Please note that PLOS ONE has specific guidelines on code sharing for submissions in which author-generated code underpins the findings in the manuscript.

We have made our code publicly available on GitHub, and included the corresponding link under the new section entitled ‘Code sharing’. Besides, in the section ‘Implementation details’, we have referenced all software libraries used for our analyses and visualizations.

3. We note that the grant information you provided in the ‘Funding Information’ and ‘Financial Disclosure’ sections do not match. When you resubmit, please ensure that you provide the correct grant numbers for the awards you received for your study in the ‘Funding Information’ section.

We have removed the funding information from the ‘Acknowledgements’ section. (Continues below).

4. Thank you for stating the following in the Acknowledgments Section of your manuscript [...]. Please remove any funding-related text from the manuscript and let us know how you would like to update your Funding Statement. Currently, your Funding Statement reads as follows: […]. Please include your amended statements within your cover letter; we will change the online submission form on your behalf.

Please change the Funding Statement to read as follows:

> *This research is supported by the Spanish State Research Agency AEI under the project S3M1P4R PID2020-115882RB-I00, as well as by the Basque Government EJ-GV under the grant ‘Artificial Intelligence in BCAM’ 2019/00432, under the strategy ‘Mathematical Modelling Applied to Health’, and under the BERC 2018–2021 and 2022–2025 programmes, and also by the Spanish Ministry of Science and Innovation: BCAM Severo Ochoa accreditation CEX2021-001142-S / MICIN / AEI / 10.13039/501100011033.*

5. Please include captions for your Supporting Information files at the end of your manuscript, and update any in-text citations to match accordingly.

As instructed, we have listed all sections, tables and figures included in our Supporting Information document, including their corresponding captions. Whenever they are mentioned in the manuscript, their references have been added.

6. We note that Supporting Figures S9-S19 in your submission contain [map/satellite] images which may be copyrighted.

We have included a dedicated section at the end of our manuscript, entitled ‘Copyright statement’. It reads:

> *Supplementary Figs S9 (geographical distribution of hospitalized cases in our cohort, in total and by COVID-19 pneumonia severity), S10–S11 (socioeconomic data), and S12–S19 (exposure to air pollution) all depict maps for data elaborated within the context of this research (see section Clinical data collection for further details). They were plotted with geopandas software [59] and public postcode polygons for Spain, hence copyright-free.*

Please let us know if any other clarification is needed.

Reviewer #1 comments:

General comment:

This paper by Hayet-Otero et al. proposes a complex mathematical model for a fully data driven approach, assessing the relevant predictors of severity in Covid-19 disease. Although this topic looks interesting, the method used to fill biofactors and clinical data into the mathematical model could result questionable, in that it did not consider some previous reports on prognostic predictors of both community acquired pneumonia (CAP) and Covid-19 disease.

We highly appreciate the time and effort invested in reviewing our manuscript. Most notably, we would like to thank you for your critical appraisal, as we believe it is indeed very constructive. You identified important limitations of our work, which in our view stem primarily from: *a)* the scarcity of data for our cohort, in particular regarding various of the biofactors you refer to (please allow us to remind that they were collected during the first wave of the pandemic); and *b)* our failure to communicate appropriately such a situation in the original report.

Please consider our discussions, in the new draft of the manuscript and below. We have done our best to address them thoroughly; with the data we have available, in our fully data-driven approach (as you remark, once again correctly).

Specific comments:

> ***1. Neutrophil-to-Lymphocyte Ratio was shown to predict intra-hospital mortality in patients with CAP (Cataudella et al., J Am Ger Soc 2017), performing better than PSI and CURB-65. This evidence further strengthened that combined, but not separate, evaluation of neutrophils and lymphocytes should be considered mandatory to improve prognosis prediction. This is due to the fact that in patients with worse prognosis neutrophilia runs in parallel with lymphopenia. It does not seem that Authors follow this strategy, since they used absolute values of both cells separately.***

We fully agree that, as you point out, there is coinciding evidence in literature about the importance of the Neutrophil-to-Lymphocyte Ratio (NLR), in both CAP and COVID-19. Interestingly, our findings also stress such importance: feature #86 (which corresponds to NLR) was the 5th most frequently selected one (Table 6, section entitled *'Feature Selection: Selected features'*).

We acknowledge that we had reported our results regarding this topic with loose text, and hence overlooked the true strength of NLR as explanatory factor. In various extracts (e.g. in the abstract and by the end of the discussion), we mentioned lymphocytes and neutrophils in absolute terms, while omitting its ratio (NLR); despite it having being ranked very high (5th ranking in Table 6), and above the two absolute values (lymphocyte, neutrophil counts: features #82, #83 in Table 6).

To correct this aspect, we included explicit mention to NLR wherever suitable, reordered the narrative list of relevant factors, and added a dedicated paragraph in 'Discussion':

> *The neutrophil-to-lymphocyte ratio (NLR) inflammatory biomarker arose, in our analyses, as one of the most informative and consistent factors to predict SARS-CoV-2 pneumonia severity. Indeed, various studies found NLR to be a strong predictor for adverse outcomes in the context of respiratory diseases (but also for sepsis, cancer, etc. [84]): from 30-day mortality in community-acquired pneumonia [85], to hazard for admission in intensive care units (ICU) due to COVID-19 [86], or hazard for COVID-19 mortality (overall and by various types of complications) [87]. In this regard, the NLR reflects two dual aspects of the immune system: innate immunity (mostly associated with neutrophils), versus adaptive immunity (mainly lymphocytes) [84]. Complementarily (but with lower ranking), the hematological counts of neutrophils and lymphocytes –addressed separately– were also among the features being selected with moderate repeatability, in alignment with the findings by [74].*

We hope that these additions contribute to clarify your concern.

> ***2. A poorer prognosis of Covid-19 patients strictly depends on an early fall in PaO2/FiO2, that was recently shown to be inversely related to NLR (Regolo et al., J Clin Med 2022). It looks as if Authors filled in the model only O2 levels. What about PaO2/FiO2?***

Thank you for another very meaningful remark. This statement is right: we were unable to provide the feature selection models with information regarding PaO_2/FiO_2 measurements.

During the stage of enrolment for our cohort (with the clinical part of our research team being under unprecedented therapeutical demand: COVID-19 1st wave), the collection of data was limited in practise. Due to this reason, we only had access to 600/1548 (38.76%) valid measurements for PaO_2, and 581/1548 (37.53%) valid measurements for PaO_2/FiO_2.

In the previous version of the draft (section *'Materials & Methods'*), we had mentioned a criterion for data quality: to discard those variables with ⩾60% missing values. However, we had omitted to report explicitly which biofactors suffered this circumstance. Following your concern, we have included such information under the epigraph *'Data collection'*, as well as in the new Fig 1 (left-most blob). These 14 dismissed variables were: SOFA score, AST, bilirubin, CPK, IL-6, BNP, troponin, ferritin, eosinophils, and platelets; as well as 4 measurements from arterial blood gas tests – pH, PaO_2, $PaCO_2$, and the PaO_2/FiO_2 ratio.

In addition, we have discussed the implications of this situation under the section *'Limitations'*.

While we fully agree with you in the sense that lacking key information about PaO_2/FiO_2 is far from desirable, we believe that the circumstances of the study were extraordinarily difficult.

3. Systemic Inflammation Index (SII), a combined index using neutrophils, platelets and lymphocytes, was shown to be another important predictor of intubation in Covid-19 patients (Muhammed et al., Pathogens 2021). Did authors consider it?

We honestly appreciate the effort of the reviewer to assess our work. From this comment, as well as from the others (both above and below), it is obvious that you have a deep knowledge of the topic. Thus, it is indeed a great honour for us to rely on such a valuable feedback.

According to the formula for *SII* introduced in the reference provided [Muhammed et al., Pathogens 2021]:

$$SII=\frac{neutrophils\cdot platelets}{lymphocytes}=NLR\cdot platelets$$

In our case, the missingness of measurements for platelets is regrettably extreme: only 71/1548 (4.59%) valid measurements. With this situation, we could at best aspire to impute platelets with a very rough estimation (possibly the mean, or the median across the cohort). In such circumstance (having imputed platelets), SII would contain the same information as NLR, up to a constant multiplicative factor.

In the revised version of our manuscript, we have mentioned this topic as well. We hope that this explanation has shed some light on the issue.

4. MECOR is an emerging score to recognize a possible viral aetiology of CAP, providing a prompt Covid-19 triage (Sambataro et al., Diagnostics 2020), helping to prevent complications.

In the case of the MECOR score, the limitations to be faced are virtually the same as for SII.

According to the formula of the score [Sambataro et al., Diagnostics 2020]:

$$MECOR=18.47-3.23\ln(leukocytes)-0.76\ln(lymphocytes)+11.94\arctan\left(\frac{monocytes\cdot neutrophils}{platelets}\right)$$

These are the percentages of valid (i.e. non-missing) data for each of the 5 biofactors involved in the definition of MECOR:

- Leukocytes: 1460/1548 = 94.32%
- Lymphocytes: 1530/1548 = 98.84%
- Monocytes: 1108/1548 = 71.58%
- Neutrophils: 1545/1548 = 99.81%
- Platelets: 71/1548 = 4.59%

Thus, to be able to estimate MECOR for our cohort, here we had to impute platelets with the median value of the training cohort (as published by Sambataro et al.): 221,000 cells/µL.

A one-way ANOVA analysis did not reveal any statistically significant difference in terms of MECOR scores across the three COVID-19 pneumonia severity groups (p=0.290), whereas a Kruskal-Wallis test (i.e. the non-parametric equivalent of ANOVA) also failed to identify statistically significant differences (p=0.268).

Most likely, this absence of conclusive findings regarding MECOR in our cohort is due to the need to impute platelets. Again, the scarcity of valid data precludes us from reaching more conclusive results.

Likewise for SII, we have discussed about this issue in the section *'Limitations'*.

> ***5. So far, Covid 19 disease is considered the result of a derangement between innate and adaptive immunity (for review, see Buonacera et al., Int J Mol Sci 2022). This is a further reason for paying attention to the strategy used in filling biofactors and clinical data into the mathematical model (see points 1,2,3)***

Thank you very much for providing this valuable and enriching insight. We have added some text addressing this topic in connection to NLR, under the epigraph *'Discussion'* (please see issue #1).

Should you find it necessary to proceed to further explanation, please let us know.

Reviewer #2 comments:

Specific comments:

1. ***Introduction section needs to be re-written to improve its quality and readability.***

After re-reading our previous draft, we agree with you in the sense that *'Introduction'* needed serious improvements. In the current version, we have re-structured the ideas, explanations and writing style in order to be more clear and concise. We hope to have matched your expectations in this regard.

2. ***The literature has to be strongly updated with some relevant and recent papers focused on the fields dealt with in the manuscript.***

Here you point out a fair query. We have now updated the *'Related works'* section with a number of recent studies which are more closely connected to the topic of FS for COVID-19 data. Besides, we have proceeded to remove some previous references which we now deem as less relevant than the new ones. Please note that various of the papers added have also been included in the *'Discussion'* section, as they support our findings.

3. ***Clinical data can be summarized in a table for better understanding.***

Thank you for pointing out this aspect. In our previous submission we had included such a table in the supplementary materials, but we failed to communicate this content properly. We have now revised and extended the explanation regarding it, more explicitly referring the reader to the appendices.

Nevertheless, in our view, the size of the table poses a major challenge as to include it in the main body of the manuscript: it now expands through 92 clinical variables and 7 full pages (Appendix B, Table S2). We believe that shortening the table, via manually picking a certain subset of them beforehand, may bring the undesired side effect of biasing the presentation of the dataset (at least to a certain extent).

Please note that Supplementary Figures S1-S7 depict the distribution of the clinical variables in our cohort; whereas Appendix B also contains other visual presentation of the information encompassed in our dataset: geographical distribution of cases, socioeconomic and pollution exposure data, etc.

In any case, let us remark that should you still consider necessary to move the table to the main body of the manuscript, we have no objection, of course.

4. ***Explain in detail what type of hyperparameter selection methodology is used for selecting best parameters for each ML algorithms.***

Very insightful comment.

As for the hyperparameters of the pre-processing pipeline, we have added a short sentence for clarification: via a preliminary exploratory stage, in which we primarily controlled for the computational burden of pre-processing (particularly, imputation).

For the FCBF filter, we acknowledge our omission in explaining the choice for δ. We have now included a mention: stating that increasing δ from its default 0 value would eventually discard almost all features, whereas decreasing it would become notably detrimental in terms of stability.

Regarding the hyperparameters of the 'wrapped' ML estimators: in an abuse of brevity, we had only stated the final resulting choices. Now we have incorporated a specific paragraph in the subsection '*Set-up of the Feature Selection algorithms*', to add up clarification. We performed cross-validated (CV) searches (grid and/or Bayesian, whenever suitable), repeated throughout bootstrap samples. Then we chose the mode of the optimal values. Note that the magnitude to maximized during the search was the geometric mean score (GMS).

Yet another important set of choices were the hyperparameters controlling the search strategies for GA and BPSO. In brief, we picked 'canonical' configurations, and then examined with how many iterations convergence could be achieved.

Since we agree fully with the reviewer about the importance of this topic, we have enhanced our supplementary materials with Appendix C, which contains 15 figures illustrating the intermediate results for

hyperparameter selection. Furthermore, we provide the reference to Hayet-Otero's MSc thesis [52] for an extended and more detailed technical report. With these, we hope to have provided sufficient detail for an appropriate understanding of our methodological choices, and for the reproducibility of the experiments.

5. *Explain why the current method was selected for the study, its importance and compare with traditional methods.*

Indeed, the manuscript was lacking an explicit statement to introduce and discuss these aspects. We have expanded accordingly our sections *'Objectives'* and *'Discussion'* (1st part) in accordance.

In brief, the differences with respect to literature are:

- ◆We do not address feature selection as an intermediate step for predictive models. Thus, we do not focus on the predictive outcomes of the selected subset, but on the properties (stability, similarity) of the FS procedure itself.
- ◆We systematically evaluate a broad range of configurations, covering all major families of FS (filters, wrappers, embedded) and with classification and regression methodologies for our ordinal classification task.
- ◆By exhaustive comparison of the selected features, we contribute to data-driven knowledge extraction about explanatory variables for SARS-CoV-2 pneumonia severity, complementing medical perspective.

6. *Authors are suggested to include more discussion on the results and also include some explanation regarding the justification to support why the proposed method is better in comparison towards other methods*

Regarding the justification of our adopted methodology and its comparison with respect to literature, we would kindly refer you to points #5 and #7.

As for the discussion of results, we rephrased various of the paragraphs, expanding important discussions about the independent supporting evidence which coincided in predictor features.

7. *Does this kind of study have never attempted before? Justify this statement and give an appropriate explanation to do so in this paper.*

To the best of our knowledge, no other published work has conducted a similar study. A new paragraph in *'Objectives'* reads as follows:

> *In this context, we deemed suitable to complement the analyses in terms of prediction capabilities by ML owing to FS. Considering that there exists a broad range of FS algorithms of different nature, here we opted for conducting an explicit and systematic comparison about the behaviour of the various FS algorithms with respect to their robustness. Indeed, this topic constitutes a field of growing importance within the ML community for understanding the FS procedure itself [12, 13], where different methods have been proposed to enhance the assessments of the stability properties of FS concerning changes in the data (see [14] for a comprehensive methodological review). In this regard and to the best of our knowledge, the approach presented here stands aside from the existing literature, as we have not identified other studies tackling the topic of robustness in FS for COVID-19 data.*

8. *There are several machine learning algorithms what is the reason for using only LR, Ridge and KNN?*

Here you raise a very insightful point, which is indeed crucial in the choice of the Machine Learning (ML) models internally used in the wrappers. We have rewritten a part of the subsection '*Set-up of the Feature Selection algorithms'* to explain our choice. In brief, we considered it was essential to be based on algorithms with few hyperparameters to tune, in order for the overall behaviour of the wrapper to be robust with respect to such choice.

LR, Ridge and KNN need a single hyperparameter: regularization strength C or α, and number k of neighbours, respectively. Comparatively: for example, a typical Support Vector Machine (SVM) would have

compelled us to select three hyperparameters: kernel function (possibly non-linear, since linear SVMs are closely linked to LR), kernel width and regularization. Other models, such as Random Forests or Multi-Layer Perceptron (MLP) neural networks pose similar challenges.

Computational simplicity played also a role. As we mentioned in item 2.II), we also performed experiments with more complex algorithms, namely Histogram-based Gradient Boosting (HGB). However, they turned out to be prohibitive in terms of computation time.

Should you find it necessary for us to provide further discussion, please let us know.

9. ***Mentioning number of features before and after using FS algorithms in table can be a simplified version of summarizing.***

Thank you for providing this suggestion, we agree that the previous version of the draft was unclear in reflecting this aspect.

As for the features prior to selection (input), in the section *'Data collection'* we have provided a disaggregation of the different numbers of features, depending on the type/source of information and the stage of pre-processing. It is complemented by: a) the new Fig 1: a flow chart across the different stages, and b) a full list of feature numbers, names, types and units in Appendix A (Table S1).

As for the features resulting after selection (denoted n_{FS} in our nomenclature), we had previously described those setups where n_{FS} had to be fixed beforehand, but we had omitted those other where n_{FS} was picked by the algorithm. To amend this, we have included the new Table 5, containing the median and 95% percentile range in all scenarios with non-fixed n_{FS}.

We hope that these additions contribute to clarify your concern.

General comment:

10. ***Overall the presentation and provided results are satisfactory.***

We would like to take this opportunity to honestly express our great gratitude for your thorough review, which has raised many important aspects that needed further explanation in our previous manuscript. We are very pleased that you found the results satisfactory, and we hope that the new version succeeds in clarifying the issues you found.

Amendments by the authors:

We took the opportunity of this revision to make a thorough examination of our draft. In addition to the very valuable issues raised by the reviewers, we noticed a small number of aspects which we felt would benefit from some degree of refinement. In any case, we believe these changes do not alter (by any means) the nature of the manuscript as to change the judgements carried out by the peer reviewers.

These changes we suggest are:

- Improved mathematical notation for the magnitudes involved in formulae (2)-(4), and (6). We had noticed minor inconsistencies in notation and lack of proper definition of certain auxiliary variables.
- A clarification on why we also address the analysis of computation times.
- Coloured versions of Figs (3)-(5), not altering their content but facilitating visualization via a colour-coded display of the plotted magnitudes.
- Removal of some secondary topics in ‘*Strengths*’, for the sake of a simplified readability of our discussion.
